# Functional paralysis of human natural killer cells by alphaherpesviruses

**DOI:** 10.1371/journal.ppat.1007784

**Published:** 2019-06-13

**Authors:** Tessa Mollie Campbell, Brian Patrick McSharry, Megan Steain, Tiffany Ann Russell, David Carl Tscharke, Jarrod John Kennedy, Barry Slobedman, Allison Abendroth

**Affiliations:** 1 Discipline of Infectious Diseases and Immunology, The University of Sydney, Sydney, New South Wales, Australia; 2 Department of Microbial Sciences, University of Surrey, Guildford, Surrey, United Kingdom; 3 John Curtin School of Medical Research, The Australian National University, Canberra, Australian Capital Territory, Australia; University of California, San Francisco, UNITED STATES

## Abstract

Natural killer (NK) cells are implicated as important anti-viral immune effectors in varicella zoster virus (VZV) infection. VZV can productively infect human NK cells, yet it is unknown how, or if, VZV can directly affect NK cell function. Here we demonstrate that VZV potently impairs the ability of NK cells to respond to target cell stimulation *in vitro*, leading to a loss of both cytotoxic and cytokine responses. Remarkably, not only were VZV infected NK cells affected, but VZV antigen negative NK cells that were exposed to virus in culture were also inhibited. This powerful impairment of function was dependent on direct contact between NK cells and VZV infected inoculum cells. Profiling of the NK cell surface receptor phenotype by multiparameter flow cytometry revealed that functional receptor expression is predominantly stable. Furthermore, inhibited NK cells were still capable of releasing cytotoxic granules when the stimulation signal bypassed receptor/ligand interactions and early signalling, suggesting that VZV paralyses NK cells from responding. Phosflow examination of key components in the degranulation signalling cascade also demonstrated perturbation following culture with VZV. In addition to inhibiting degranulation, IFN-γ and TNF production were also repressed by VZV co-culture, which was most strongly regulated in VZV infected NK cells. Interestingly, the closely related virus, herpes simplex virus type 1 (HSV-1), was also capable of efficiently infecting NK cells in a cell-associated manner, and demonstrated a similar capacity to render NK cells unresponsive to target cell stimulation–however HSV-1 differentially targeted cytokine production compared to VZV. Our findings progress a growing understanding of pathogen inhibition of NK cell function, and reveal a previously unreported strategy for VZV to manipulate the immune response.

## Author summary

Natural killer (NK) cells–as their name implies–are the immune system’s ready to respond ‘killers’, being able to help control viral infection by cytolytic killing of infected cells and secretion of pro-inflammatory cytokines to activate and direct the immune response. In retaliation, viruses like varicella zoster virus (VZV; the cause of chickenpox and shingles) work to dampen the immune system in order to establish infection in human hosts. We have identified a previously uncharacterised ability of VZV to render NK cells unresponsive to target cells, hindering NK cells from both cytotoxic function and cytokine production. NK cells still maintained predominantly stable expression of functional surface receptors, and were capable of releasing cytotoxic granules when given a receptor-independent stimulus. In this way, VZV paralyses NK cells from functionally responding to target cells, essentially taking the ‘killer’ out of natural killer cells.

## Introduction

Natural killer (NK) cells are cytotoxic innate lymphocytes that exert a significant influence on the control of viral infection. Predominantly residing in peripheral blood circulation, lymphoid tissue and the liver, NK cells detect infected or aberrantly transformed cells [1]. Sensing of cells is achieved by a diversity of activating and inhibitory receptors through which the integration of signals stringently regulates the NK cell response [2, 3]. When sufficient activation is reached, signals from the activating receptors proceed to downstream signalling pathways culminating in polarised release of preformed granules containing perforin and granzymes [4, 5]. These cytotoxic granules will induce apoptosis of the target cell, thereby mediating the cytolytic killing function of NK cells. In a secretion process distinct from degranulation, NK cells will additionally release potent pro-inflammatory cytokines, such as interferon-γ (IFN-γ) and tumour necrosis factor (TNF) [6].

The function of NK cells is tailored to directly control viral infection and contribute to promotion and immunoregulation of host defence [7]. In response to this, many viruses, including herpesviruses, encode strategies to evade detection and elimination by NK cells [8, 9]. This is particularly the case with herpesviruses, where their lifelong persistence depends upon a balance between immune evasion and control that has arisen due to millennia of coevolution between virus and host [10, 11]. A clear example of this is seen with infections caused by the human alphaherpesvirus varicella zoster virus (VZV), which presents as varicella during primary infection and herpes zoster following viral reactivation from latency. In studies of NK cell deficiency, it has been reported that many of these patients are highly susceptible to severe, disseminated varicella [12–16], indicating an essential requirement for effective NK cell immunity against VZV. To circumvent this immune control, however, we have shown that VZV manipulates expression of ligands detected by the activating NKG2D receptor of NK cells [17]. Significantly, we found there was limited activation of NK cells in response to VZV infected target cells *in vitro*, suggesting substantial modulation of the surface of VZV infected cells to reduce NK cell detection of infection [17]. Additionally, we and others have demonstrated that another human alphaherpesvirus, herpes simplex virus type 1 (HSV-1), can also downregulate expression of NKG2D ligands [17–19], while for HSV-2 it has been shown that the virus can evade NK cell activity by reducing expression of CD112– the ligand to NK cell activating receptor DNAM-1 [20]. These studies demonstrate that human alphaherpesviruses encode several strategies to impede NK cell detection of virally infected cells, however there has been little investigation into how these viruses may directly affect NK cells and their function.

More recently, we have identified that peripheral blood CD56^dim^ NK cells are highly permissive to VZV infection [21], suggesting that VZV may use these immune cells to aid dissemination of virus throughout the host. Given the discovery of this novel tropism, we investigated whether VZV infection of primary human NK cells altered their functional activity. Using a unique co-culture system that more closely models *in vivo* interactions, we cultured human peripheral blood mononuclear cells (PBMCs) with VZV infected cells, and then assessed NK cell functional capacity. Our findings provide the first evidence that co-culture of NK cells with VZV mediates a profound paralysis of cytolytic activity, affecting both VZV infected NK cells as well as NK cells exposed to VZV. Comparative experiments with HSV-1 remarkably revealed a shared capacity for this alphaherpesvirus to also infect and inhibit NK cell function. Furthermore, culture with VZV inhibited NK cell production of both IFN-γ and TNF cytokines, which was in contrast to HSV-1 where only IFN-γ expression by infected NK cells was blocked. These findings indicate for the first time that multiple aspects of NK cell anti-viral function are directly targeted for immune evasion by two human alphaherpesviruses.

## Results

### VZV culture renders NK cells unresponsive to target cell stimulation

**Fig 1:**
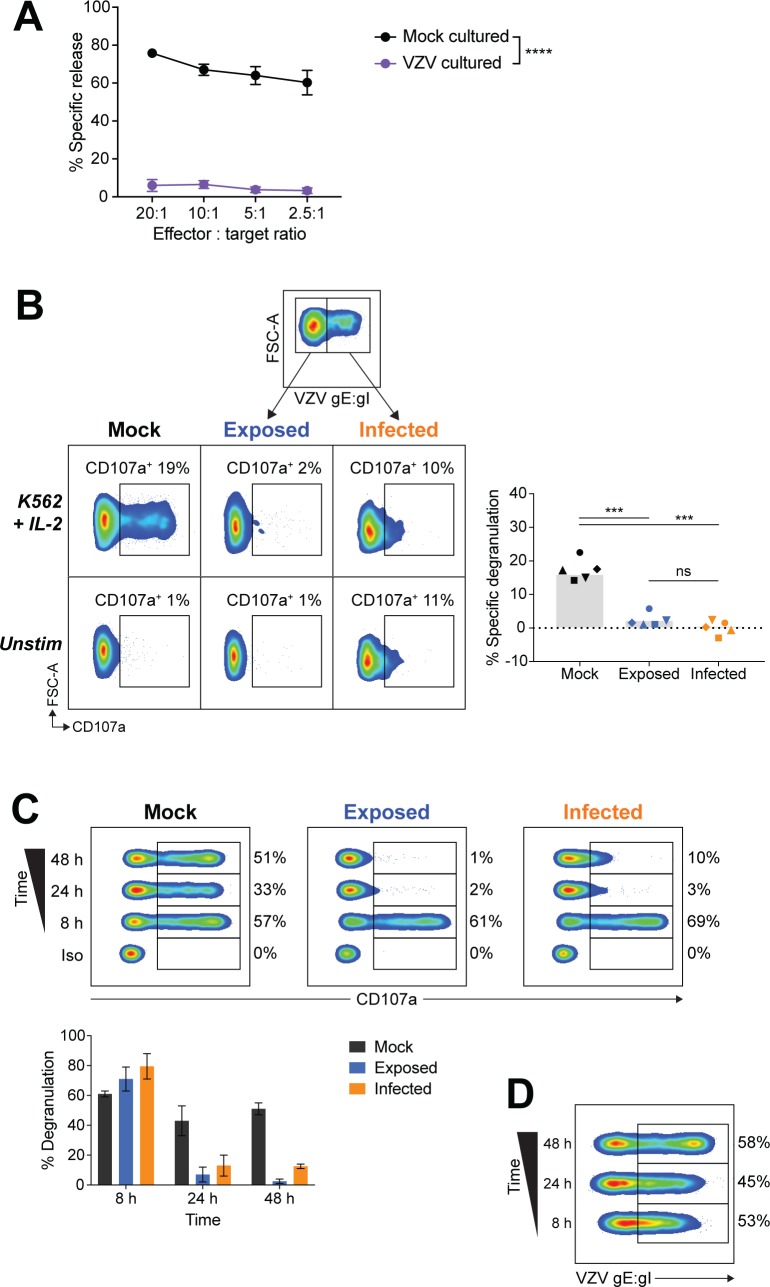
NK cells cultured with VZV are unresponsive to K562 stimulation. (A) NK cells (CD3^–^CD56^+^) were FACS sorted from CD56^+^-selected lymphocytes following culture with mock or VZV inoculum, plus 200 U/ml IL-2. At 2 days pi isolated NK cells were then challenged with K562 cells in a calcein-AM release assay. Graph depicts means ± SEM for four donors. ****P < 0.0001 (repeated measures two-way ANOVA with Sidak correction). (B) Flow cytometry of degranulation (CD107a^+^) of NK cells (viable CD3^–^CD56^+^ cells) from PBMCs cultured with mock or VZV inoculum for 2 days, and stimulated with K562 cells with IL-2 or left unstimulated. VZV exposed or infected was determined by surface staining for VZV gE:gI. Graph shows frequency of specific degranulation against K562 cells for five donors. Symbols represent individual donors, and grey columns indicate mean. ***P < 0.001, ns = not significant (repeated measures one-way ANOVA with Sidak correction). (C) Flow cytometry of degranulation (CD107a^+^) or isotype control stain (Iso) of NK cells (viable CD3^–^CD56^+^ cells) from PBMCs mock cultured, exposed to VZV, or VZV infected for the times indicated, and stimulated with K562 cells with IL-2. Graph depicts mean frequency of degranulation (± SEM) for two donors. (D) Flow cytometry of VZV infection (gE:gI^+^) of NK cells from (C).

To investigate the effect of VZV on NK cell cytolytic function, we examined lysis of the K562 erythroleukemia cell line–a classic NK cell target that is readily lysed due to a lack of major histocompatibility complex class I (MHC I) expression. Our effector NK cells were obtained from CD56^+^-selected lymphocytes from human PBMCs, which were then cultured with mock or VZV infected cell inoculum in the presence of 200 U/ml interleukin-2 (IL-2) for 1 day. We have previously demonstrated that this technique results in 30%–85% of NK cells being VZV infected [21]. In the current experiments we observed infection of 44–73% of NK cells, as determined by flow cytometry detection of VZV surface glycoprotein heterodimer glycoprotein E: glycoprotein I (gE:gI) at 1 day post infection (pi). CD3^–^CD56^+^ NK cells were then isolated by FACS sorting and rested overnight before, at 2 days pi, NK cells were challenged with K562 target cells at varying effector to target cell ratios in a calcein-AM release assay (Fig 1A). As expected, mock NK cells efficiently lysed K562 target cells. In contrast, VZV cultured NK cells were almost completely incapable of lysing target cells even at a 20:1 effector to target ratio, where only 6% of K562 cells were lysed compared to 75% lysis achieved by mock NK cells.

VZV cultured NK cells would include both infected NK cells and NK cells that were exposed to the viral inoculum but had not progressed to productive infection. To delineate whether the loss of cytolytic function occurred only within the VZV infected NK cells, we examined NK cell function by flow cytometry which allowed concurrent staining for VZV gE:gI to differentiate VZV infected (gE:gI^+^), from VZV exposed (gE:gI^–^), NK cells. PBMCs were co-cultured with mock or VZV infected cell inoculum for 2 days, before addition of K562 targets and subsequent examination of degranulation by flow cytometry detection of CD107a. As cytolytic granules are lined with CD107a protein, staining of cell-surface CD107a allows measurement of active degranulation. Gating on live NK cells (CD3^–^CD56^+^ cells) we surprisingly observed a significant reduction in degranulation against K562 targets by both infected and exposed NK cells, compared to mock cultured NK cells (Fig 1B). As a higher level of non-specific degranulation was observed in infected NK cells, specific degranulation was calculated as degranulation against K562 targets minus background degranulation when unstimulated (Fig 1B, right). From this analysis, it was clear that VZV is able to inhibit both infected and exposed NK cells from responding to target cell stimulation.

To determine the kinetics of NK cells becoming refractory to degranulation by VZV culture, we performed a time course of VZV infection. PBMCs were cultured with mock or VZV inoculum for varying lengths of time before stimulation with K562 targets for 5 hours so that at the end of the assay NK cells had been infected for 8, 24 or 48 hours. Flow cytometry detection of CD107a revealed that VZV inhibited both exposed and infected live NK cells by 24 hours pi (hpi) (Fig 1C). 8 hours of co-culture, however, was not sufficient time to mediate inhibition to target cell stimulation, despite the fact that VZV infection of live NK cells was established within this timeframe (Fig 1D).

Taken together these findings demonstrate that VZV culture has a powerful ability to drastically reduce NK cell cytolytic function against K562 target cells.

### VZV cultured NK cells degranulate with PMA/I stimulation but display impaired IFN-γ and TNF production

**Fig 2:**
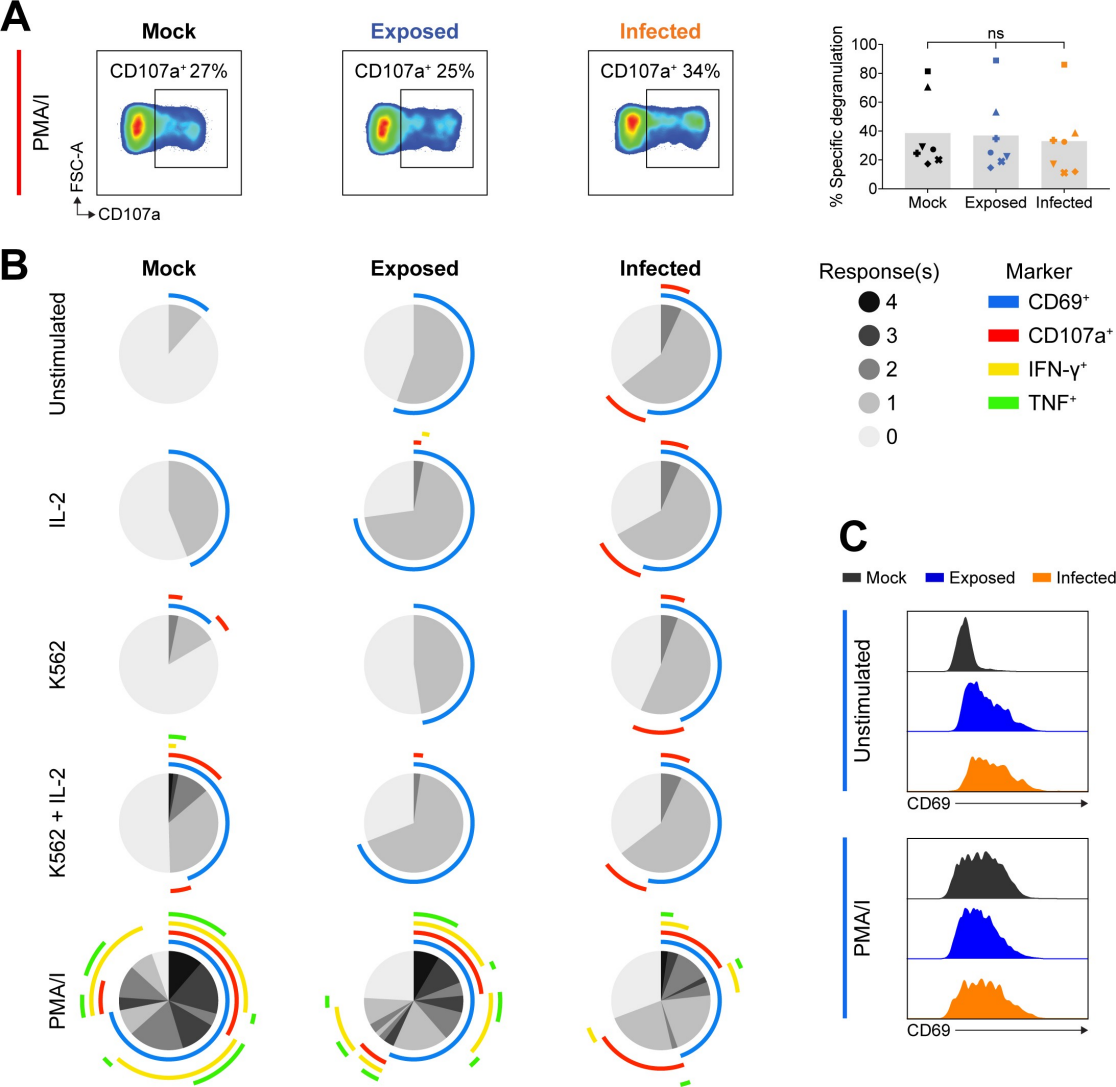
Polyfunctional analysis of VZV cultured NK cells. PBMCs were mock cultured, exposed to VZV, or VZV infected for 2 days and stimulated as specified for flow cytometry analysis of NK cells (viable CD3^–^CD56^+^ cells). (A) Degranulation (CD107a^+^) following stimulation with PMA and ionomycin (PMA/I). Graph shows specific degranulation with PMA/I stimulation for seven donors. Symbols represent individual donors, and grey columns indicate mean. ns = not significant (Friedman test with Dunn’s correction). (B) SPICE pie charts show the proportion of responses to different stimuli (listed left) based on combinations of detected CD69, CD107a, IFN-γ and TNF expression. Pie slices indicate the number of responses (0–4) (key, middle right). Arcs detail which markers were detected for each response (key, outer right). Data represent the means of three donors. (C) Histograms show CD69 expression for NK cells either unstimulated (above) or stimulated with PMA/I (below), for one representative donor (n = 6 donors).

To investigate how VZV culture inhibited cytotoxicity against target cells, we stimulated NK cells with a lymphocyte activation cocktail consisting of phorbol myristate acetate and ionomycin (referred to as PMA/I). In contrast to target cell stimulation, PMA/I stimulates degranulation from a midpoint in the signalling pathway, bypassing the initial receptor/ligand interactions that initiate degranulation against a target cell [22]. We cultured PBMCs with mock or VZV inoculum for 2 days and then stimulated with PMA/I for 5 hours. Subsequent flow cytometry analysis of live NK cell CD107a expression revealed that exposed and infected NK cells degranulated to similar levels as mock cultured NK cells when stimulated with PMA/I (Fig 2A). This finding indicates that for VZV exposed and infected NK cells, their degranulation machinery is still intact and they are capable of degranulating, thus implying that there is a specific block upstream in the pathway to degranulation, leading to inhibition of the response to K562 cells.

Given the difference in VZV cultured NK cell degranulation between PMA/I and K562 stimulation, we were interested in examining other NK cell responses, such as cytokine production. Specifically, we measured degranulation by CD107a detection, CD69 (a marker of activation), and the cytokines IFN-γ and TNF by flow cytometry, following stimulation of NK cells cultured with mock or VZV inoculum for 2 days. In order to examine polyfunctional NK cell responses, multiparameter flow cytometry was paired with SPICE (Simplified Presentation of Incredibly Complex Evaluations) analysis, which allowed easy visualisation and comparison of the variety of responses from mock, exposed and infected NK cells evoked by different stimuli (Fig 2B). When unstimulated, VZV cultured live NK cells showed higher CD69 expression compared to mock (Fig 2B and 2C), suggesting that NK cells were activated in response to VZV, paralleling similar reports for VZV infected T cells [23]. Upon PMA/I stimulation, all NK cells showed similar levels of enhanced activation as determined by increased CD69 expression (Fig 2B and 2C).

For mock NK cells, stimulation with IL-2, or K562s without pre-activation with IL-2, elicited minimal response; however, challenge with K562s plus IL-2 stimulated degranulation and modest IFN-γ and TNF production (Fig 2B). In contrast, exposed and infected live NK cells showed not only reduced specific degranulation against K562s, but also a lack of cytokine expression (Fig 2B). This indicates that VZV culture inhibits both degranulation and cytokine expression in response to K562 stimulation, suggesting VZV culture renders NK cells completely functionally unresponsive to target cells.

**Fig 3:**
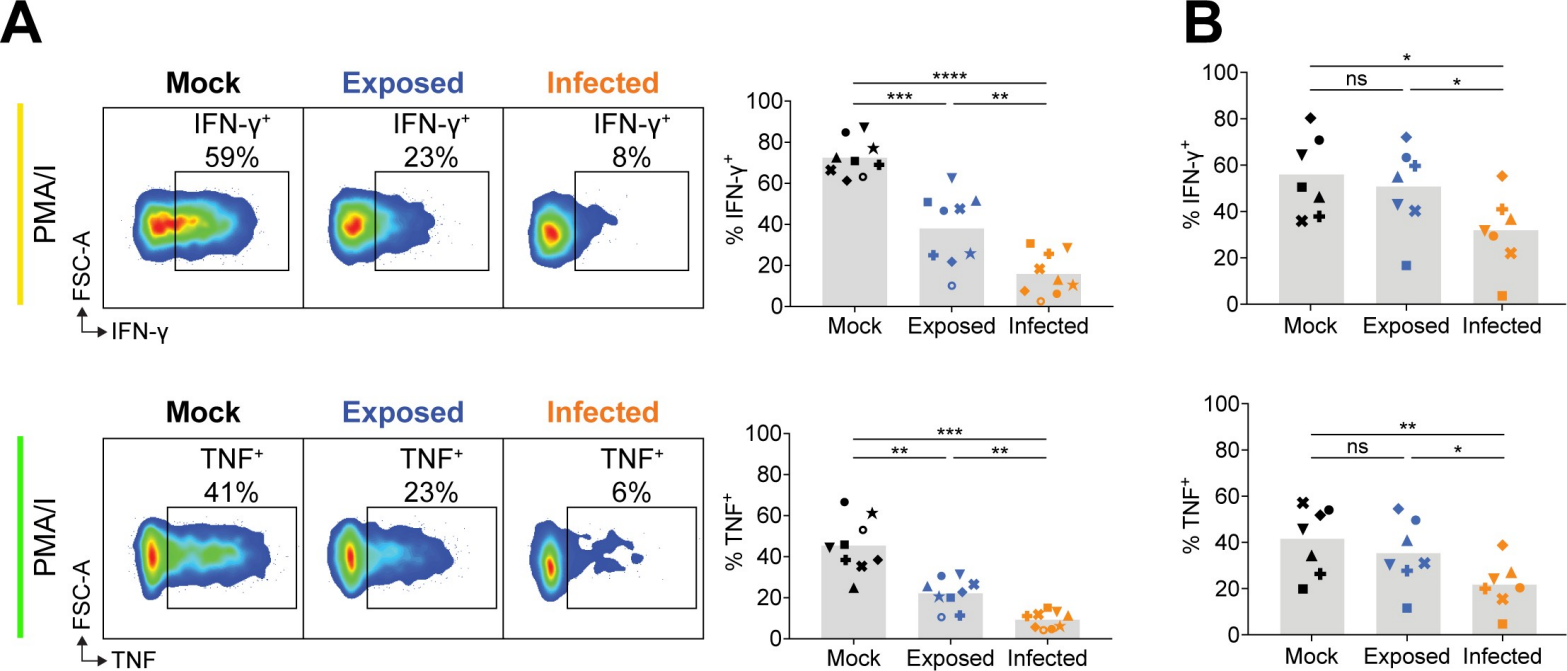
VZV infected NK cells produce less IFN-γ and TNF upon PMA/I stimulation. PBMCs were mock cultured, exposed to VZV, or VZV infected for 2 days (A) or 1 day (B) and stimulated with PMA/I for flow cytometry analysis of IFN-γ (top panels) or TNF (bottom panels) expression by NK cells (viable CD3^–^CD56^+^ cells). Symbols represent individual donors, and grey columns indicate mean. *P < 0.05, **P < 0.01, ***P < 0.001, ****P < 0.0001, ns = not significant (Repeated measures one-way ANOVA with Tukey correction (A) or Friedman test with Dunn’s correction (B)). Data are from nine donors (A) or seven donors (B).

As additionally demonstrated in Fig 2A, SPICE analysis revealed that PMA/I stimulation lead to similar levels of degranulation for mock, exposed and infected NK cells (Fig 2B). However, it was apparent that the degree of cytokine production differed across the NK cell populations following stimulation with PMA/I. Thus, examining cytokine expression more closely at 2 days pi, we found IFN-γ and TNF expression in exposed NK cells was significantly diminished, and in infected NK cells the reduction of cytokines produced was even further pronounced with a 4–5-fold reduction compared to mock (Fig 3A). At 1 day pi, however, only infected NK cells were significantly affected in their production of IFN-γ and TNF (Fig 3B).

Collectively, functional interrogation with different stimuli indicated that while exposed and infected NK cells exhibited equal specific inhibition of cytolytic function against target cells, VZV infected NK cells were more significantly impaired in their ability to produce IFN-γ and TNF.

### Following VZV culture the NK cell surface receptor repertoire is predominantly stable

The observation that VZV cultured NK cells could degranulate with PMA/I treatment, but did not respond to external stimulation with K562 cells raised the possibility that the inhibition of function could be attributed to a loss of cell-surface receptor expression required for NK cell function. Using multiparameter flow cytometry we assessed a broad repertoire of receptors including activating NK cell receptors CD160, NKG2D, NKG2C, the natural cytotoxicity receptors (NKp46, NKp44 and NKp30), and NKp80; co-receptors for activation DNAM-1 and 2B4; direct apoptosis stimulator TNF-related apoptosis-inducing ligand (TRAIL); inhibitory NK cell receptor BTLA; adhesion molecule TACTILE; and the CD11a subunit of cellular adhesion integrin, lymphocyte function-associated antigen 1 (LFA-1). As potent inhibition of degranulation was readily observed after 1 day of VZV co-culture (Fig 1), we examined NK cells (live CD3^–^CD56^+^ cells) from PBMCs cultured with mock or VZV inoculum for 1 day, in the presence or absence of 200 U/ml IL-2. Comparison with IL-2 treatment was included as the degranulation assays against K562 cells were performed with concurrent IL-2 stimulation.

**Fig 4:**
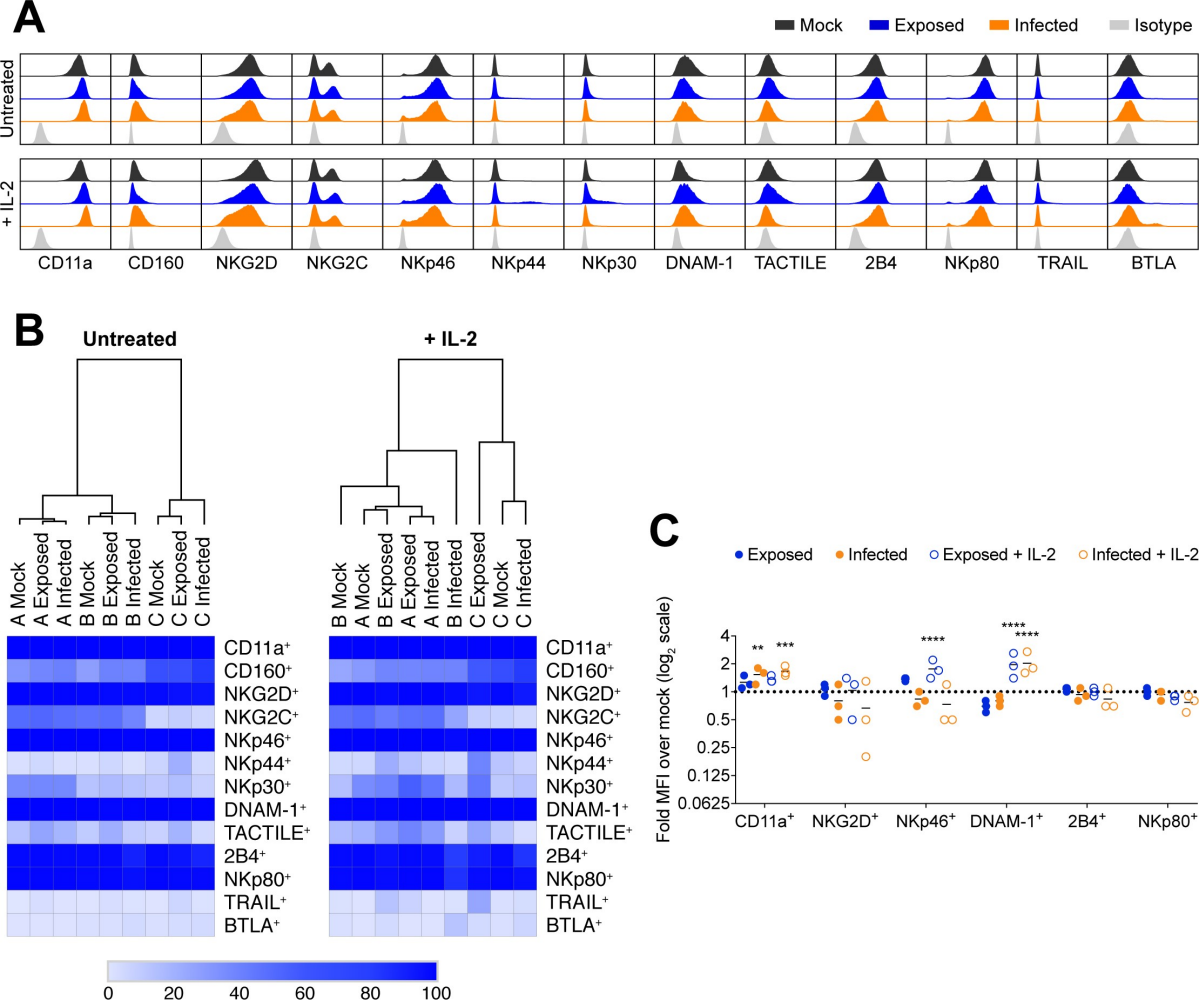
Profiling of cell surface receptor phenotype of VZV cultured NK cells. PBMCs were mock cultured, exposed to VZV, or VZV infected untreated or with 200 U/ml IL-2 for 1 day. NK cells (viable CD3^–^CD56^+^ cells) were assessed by flow cytometry for cell surface receptor expression (A), and heatmaps show receptor expression as measured by percentage positive with hierarchical clustering for 3 donors (denoted A, B and C) (B). (C) Graph shows fold change over mock in median fluorescence intensity (MFI) for ubiquitously expressed receptors (n = 3). Symbols represent individual donors. Dotted line at y = 1 indicates point of variance from mock. Statistical analysis performed compared to mock. **P < 0.01, ***P < 0.001, ****P < 0.0001 (repeated measures two-way ANOVA with Dunnett’s correction).

Flow cytometry analysis of cell-surface receptor expression appeared predominantly consistent between mock, exposed and infected NK cells (Fig 4A). To examine modulation of the cell-surface profile collectively–rather than each receptor individually–we visualised the frequency of expression of each marker by heatmap and performed hierarchical clustering across the 3 donors evaluated (Fig 4B). For untreated conditions, the dendrogram indicated that the PBMC donor was a more significant contributor to variation in the cohort, than VZV exposure or infection. With IL-2 stimulation, more variation in receptor expression was observed; however, changes associated with infection or exposure were not sufficiently consistent to cluster together for all donors.

Additional analysis of median fluorescence intensity (MFI) was calculated to evaluate changes in intensity of expression for cell-surface receptors that were homogenously expressed on the majority of NK cells (Fig 4C). Compared to mock NK cells, we observed a significant increase in CD11a density on infected NK cells (+/–IL-2), increased expression of NKp46 on exposed NK cells when treated with IL-2, and increased DNAM-1 on the surface of both exposed and infected NK cells with IL-2 treatment. As NKG2D and NKp46 are key receptors involved in detection of K562 cells [24, 25], we examined expression of these two receptors in six additional donors. Together with the three donors depicted in Fig 4, we found VZV infection in the presence of IL-2 to significantly downregulate NKG2D surface expression (S1A Fig), while NKp46 expression was significantly upregulated in exposed NK cells but reduced with infection (S1B Fig). The range of modulated receptor expression, as well as the contrasting differences between exposed and infected NK cells, suggests that changes in expression of NKG2D and NKp46 are unlikely to explain the observed loss of cytolytic function.

Considering the cumulative analysis of receptor expression, the NK cell receptor profile is remarkably stable following culture with VZV. When NK cells were first CD56^+^-selected before co-culture with mock or VZV inoculum (as they were in the calcein-AM release assay in Fig 1A), the trend of receptor expression was also consistent (S2 Fig). These findings suggest that there is not sufficient modulation of receptor expression to explain the loss of function seen in VZV cultured NK cells. The premise that downregulation of a particular receptor is not responsible for functional inhibition is further supported by stimulation experiments using phytohaemagglutinin (PHA). PHA crosslinks glycoproteins on the cell surface, however while this led to degranulation of mock NK cells, VZV exposed and infected NK cells displayed inhibited degranulation (S3 Fig), implying that broad ligation of cell-surface receptors could not stimulate degranulation of VZV cultured NK cells.

Considered together, the data thus far indicated that VZV cultured NK cells were capable of degranulating, but no longer responded to target cell stimulation either cytolytically or with cytokine production. Furthermore, the reason for this inhibition of functional responsiveness did not appear to be mediated by a loss of cell-surface receptor expression.

### NK cell function is comparably inhibited by herpes simplex virus

**Fig 5:**
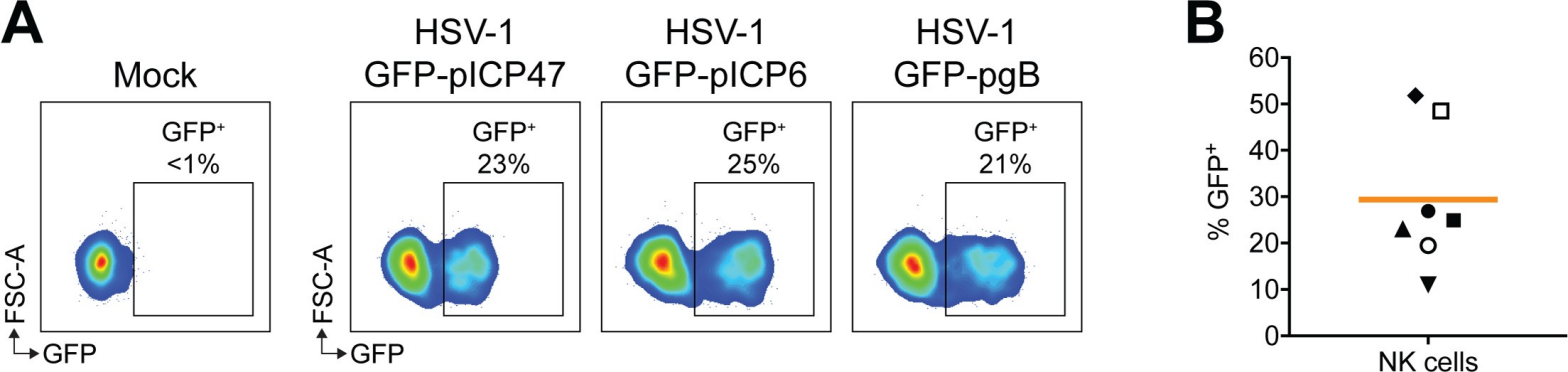
HSV-1 infects human NK cells. (A) PBMCs were cultured with mock infected inoculum or inoculum infected with HSV-1 expressing GFP under different viral promoters (ICP47, ICP6 or gB as specified) for 1 day. Infection of NK cells (viable CD3^–^CD56^+^ cells) was determined by flow cytometry detection of GFP. (B) Frequency of NK cell infection with HSV-1 GFP-pICP47 performed as in (A), for seven donors. Symbols represent individual donors, and bar indicates mean.

VZV is closely related to herpes simplex virus type 1 (HSV-1), with both being human alphaherpesviruses. As these viruses are distinct in the diseases they cause, we asked whether HSV-1 also had the capacity to modulate NK cell function. Previous studies have suggested that human NK cells may be permissive to HSV-1 infection [26, 27]. To investigate cell-associated HSV-1 infection of NK cells, we cultured human PBMCs with mock infected inoculum or inoculum infected with GFP-expressing HSV-1. Three viruses were used in infection experiments that were engineered to express GFP under an immediate early (ICP47; infected cell protein 47), early (ICP6) or late (gB; glycoprotein B) viral promoter [28]. At 1 day pi, NK cells were assessed for GFP expression by flow cytometry, and we observed similar levels of GFP^+^ cells with all three viruses (Fig 5A and 5B), indicating that HSV-1 was capable of infecting live NK cells, and undergoing the full temporal cascade of viral gene expression.

**Fig 6:**
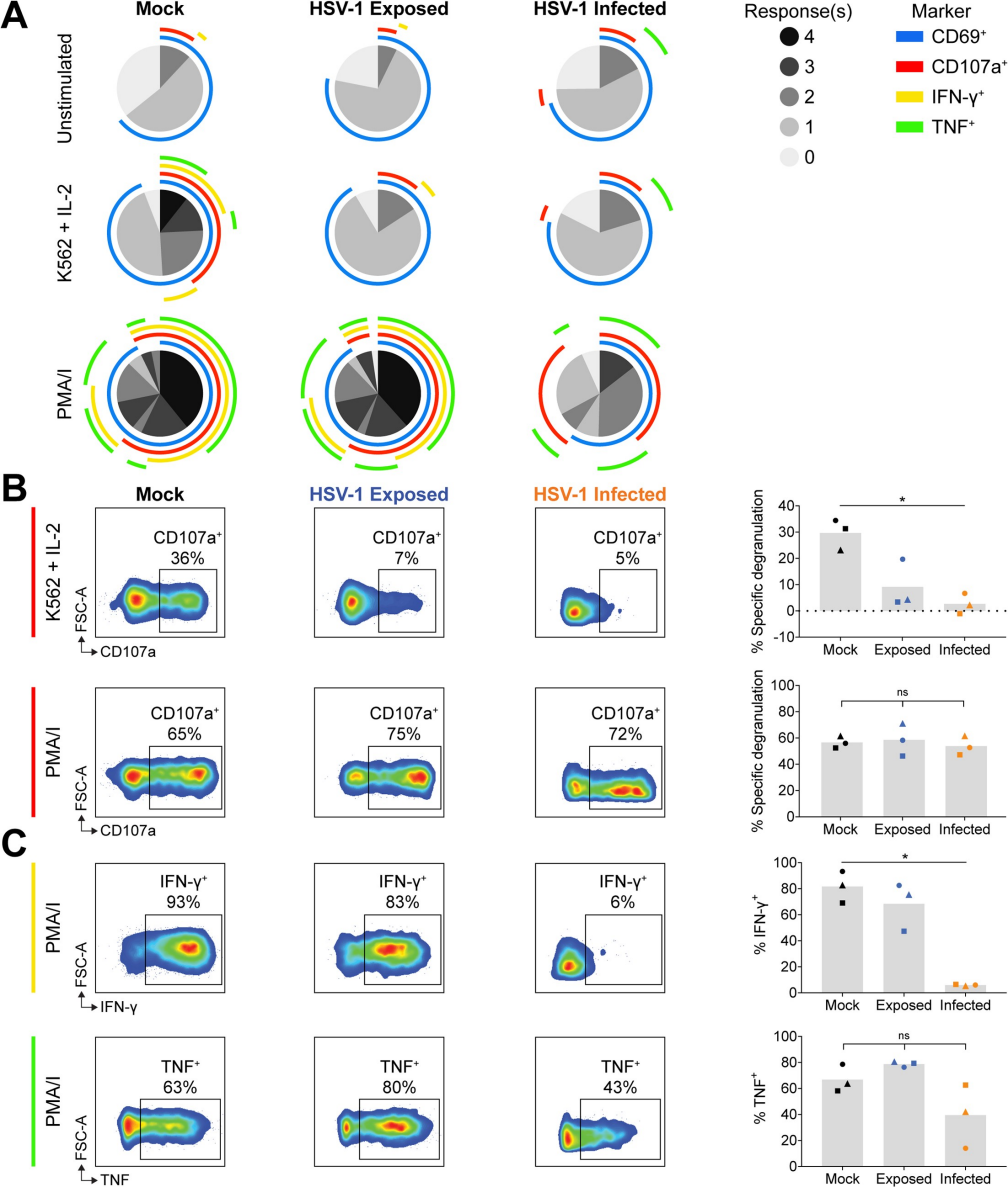
HSV-1 culture of NK cells reduces responsiveness to K562 stimulation and infection blocks IFN-γ expression. PBMCs were mock cultured, exposed to HSV-1, or HSV-1 infected for 1 day and stimulated as specified for flow cytometry analysis of NK cells (viable CD3^–^CD56^+^ cells). (A) SPICE pie charts show the proportion of responses to different stimuli (listed left) based on combinations of detected CD69, CD107a, IFN-γ and TNF expression. Arcs detail which markers were detected for each response (key right). Data represent the means of three donors. (B) Degranulation (CD107a^+^) following stimulation with K562 cells with IL-2 (top panel) or PMA/I (bottom panel). Graphs show specific degranulation for specified stimulus for three donors. (C) IFN-γ and TNF expression following stimulation with PMA/I. Graphs show frequencies of positive cells for three donors. Symbols represent individual donors, and grey columns indicate mean. *P < 0.05, ns = not significant (Friedman test with Dunn’s correction).

In direct comparison to VZV, we cultured PBMCs with mock or HSV-1 inoculum for 1 day, and challenged NK cell functional capacity by PMA/I or K562 target with IL-2 stimulation. SPICE and conventional flow cytometry analysis revealed that, similar to VZV, both HSV-1 exposed (GFP^–^) and infected (GFP^+^) live NK cells had reduced degranulation against K562 target cells, despite maintained degranulation with PMA/I stimulation (Fig 6A and 6B). In contrast to VZV, however, HSV-1 culture did not significantly affect TNF production with PMA/I treatment. IFN-γ production was also not affected in HSV-1 exposed NK cells, however in infected NK cells, IFN-γ expression was dramatically reduced 14–fold compared to mock. Thus, HSV-1 differentially regulates cytokine production compared to VZV.

### VZV inhibition of NK cell degranulation is contact-dependent

**Fig 7:**
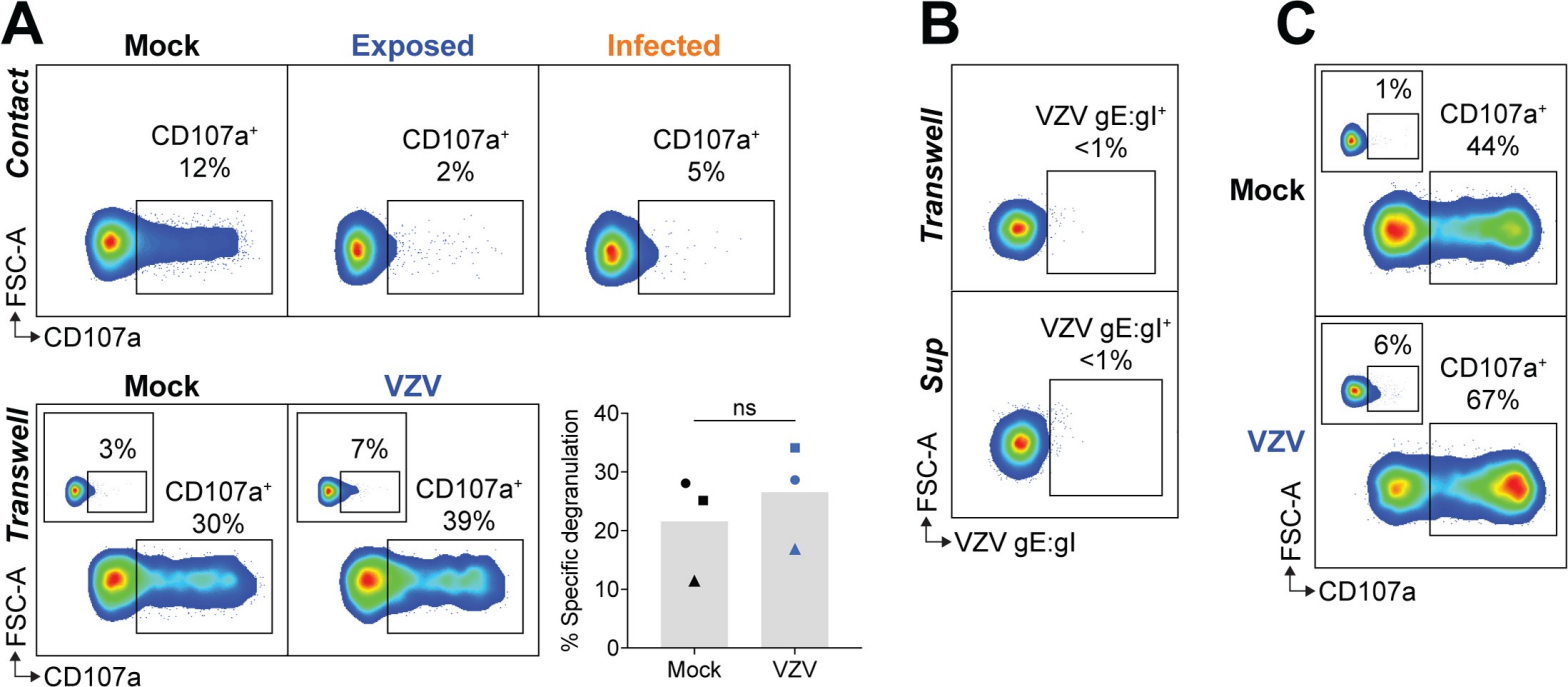
VZV inhibition of NK cell function is contact-dependent. (A) PBMCs were either mock cultured, exposed to VZV, or VZV infected (top panel), or separated from the inoculum by a Transwell membrane (bottom panel) for 1 day. PBMCs were challenged with K562 cells with IL-2 or left unstimulated (inset panels) and NK cells (viable CD3^–^CD56^+^ cells) assessed for degranulation (CD107a^+^) by flow cytometry. Graph shows specific degranulation against K562 cells for three donors. Symbols represent individual donors, and grey columns indicate mean. ns = not significant (two-tailed Wilcoxon test). (B) PBMCs were either separated from VZV inoculum by a Transwell membrane or treated with supernatant harvested from PBMCs cocultured with VZV inoculum. Flow cytometry plots show surface staining for VZV gE:gI on NK cells (viable CD3^–^CD56^+^ cells). (C) PBMCs were treated for 1 day with supernatant harvested from PBMCs cocultured with VZV inoculum, and challenged with K562 cells with IL-2 or left unstimulated (inset panels) and NK cells (viable CD3^–^CD56^+^ cells) assessed for degranulation (CD107a^+^) by flow cytometry. Data are representative of n = 3 donors (Transwell separated) (A & B) or n = 2 donors (supernatant treated) (B & C).

The finding that both VZV exposed and infected NK cells were inhibited in their cytolytic function suggested the possibility that inhibition was mediated through the culture supernatant. To test this, PBMCs were separated from mock or VZV inoculum by a Transwell membrane, before stimulation with K562 targets and flow cytometry detection of CD107a. In contrast to NK cells in direct contact with VZV inoculum, Transwell-separated NK cells were not inhibited in their degranulation against K562s (Fig 7A). In the Transwell system, PBMCs did not become infected with VZV as determined by the absence of VZV gE:gI staining (Fig 7B). This was expected as cell-to-cell contact is required for infection due to the highly cell-associated nature of VZV *in vitro*, whereby virus is not actively released into the culture supernatant [29]. It was therefore possible that an inhibitory soluble factor may be secreted from infected PBMCs, which were not present in the Transwell system. To investigate this, supernatant from 1 day pi co-cultures of PBMCs in direct contact with mock or VZV inoculum were harvested and cultured with fresh PBMCs for 1 day which were then challenged with K562 stimulation. Analysis of CD107a expression revealed that NK cells treated with the VZV co-culture supernatant degranulated just as efficiently as NK cells with mock co-culture supernatant (Fig 7C). Culture of PBMCs with the supernatant also did not lead to VZV infection, as expected (Fig 7B).

Taken together, the results in Fig 7 demonstrate that the inhibition of NK cell degranulation observed is not mediated by a soluble factor, but rather direct contact between NK cells and VZV inoculum is required for VZV to exert its inhibitory effect. To delineate whether the inhibition was mediated by the infected ARPE-19 inoculum cells or the virus directly, functional assays were performed using extracted cell-free VZV supernatants. PBMCs were co-cultured with mock or VZV cell-free preparations for 1 day in the presence of IL-2, and then challenged with K562 target cells. Measurement of gE:gI staining revealed infection of NK cells by cell-free VZV, but at a lower efficiency of infection than that achieved by cell-associated VZV transmission (S4A Fig), in accordance with our previous work [21]. When CD107a was examined, it was observed that NK cells which were infected or exposed to cell-free VZV had reduced degranulation against K562s, compared to mock cultured NK cells (S4B and S4C Fig). This finding suggests that the inhibition of NK cell function by VZV is directly mediated by the virus. In support, functional assays performed with VZV inoculum that had been UV-irradiated or fixed with 1% formaldehyde no longer conferred an inhibitory phenotype to co-cultured NK cells (S5 Fig). The lack of NK cell inhibition observed with inactivated inoculum suggests that the surface expression of VZV-infected cells is not responsible for paralysing NK cell function, but rather that active and functionally-intact VZV is required to mediate this effect.

### VZV culture reduces phospho–SLP-76 levels and increases ERK1/2 phosphorylation

**Fig 8:**
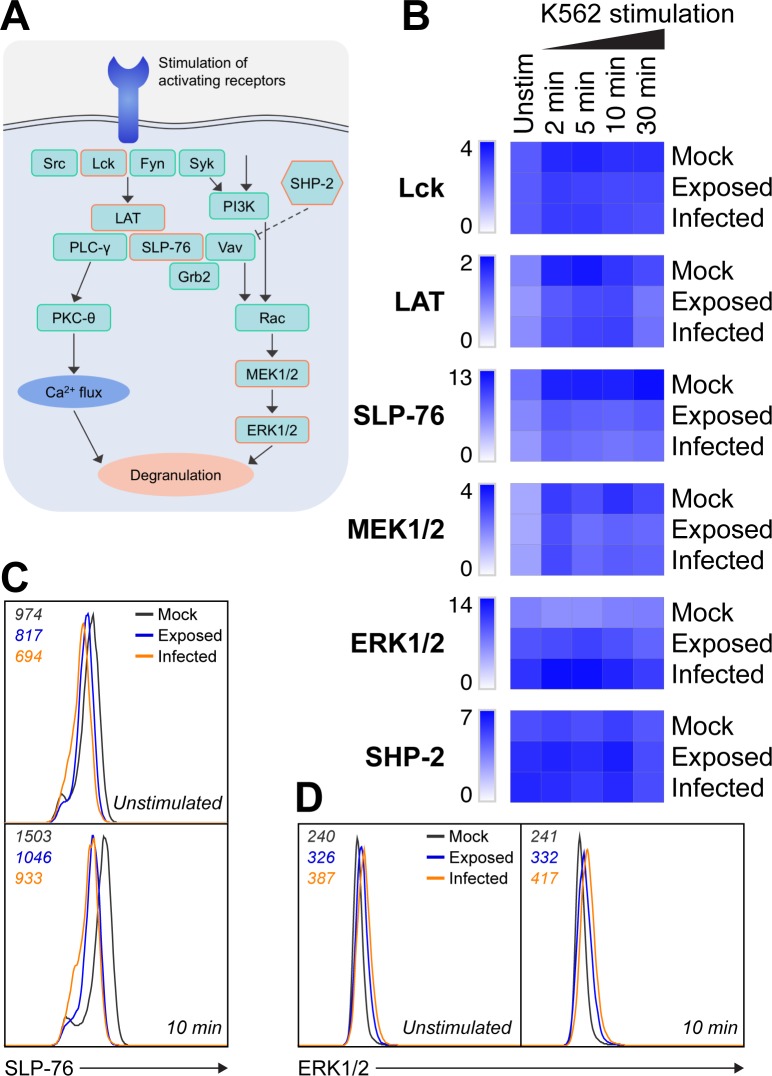
VZV culture interferes with NK cell signalling. (A) Simplified diagram of the general NK cell signalling pathway leading to degranulation, as well as the inhibitory action of SHP-2. Examined signalling molecules are indicated with an orange outline. (B-D) PBMCs were mock cultured, exposed to VZV, or VZV infected in the presence of 200 U/ml IL-2 for 1 day and either left unstimulated or stimulated with K562 cells for 2, 5, 10 or 30 min as specified. Phosphorylation of signalling markers in NK cells (CD3^–^CD56^+^ cells) was detected by flow cytometry. (B) Heatmap of phosphorylated signalling marker median fluorescence intensity (MFI) fold increase. (C) Histogram shows phospho–SLP-76 expression for NK cells unstimulated (above) and after 10 min stimulation with K562 cells (below). (D) Histogram shows phospho–ERK1/2 expression for NK cells unstimulated (left) and after 10 min stimulation with K562 cells (right). MFI values are indicated on the top left of the histogram. Data are representative of three donors. ERK1/2, extracellular signal-regulated kinase 1 and 2; Grb2, growth factor receptor-bound protein 2; LAT, linker for activation of T cells; MEK1/2, mitogen-activated or extracellular signal-regulated protein kinase kinase 1 and 2; PI3K, phosphatidylinositol 3-kinase; PKC-θ, protein kinase C-θ; PLC-γ, phospholipase C-γ; SHP-2, Src homology phosphatase 2; SLP-76, SH2 domain-containing leukocyte phosphoprotein of 76 kD.

Considering that VZV cultured NK cells predominantly maintained cell-surface receptor expression and were capable of degranulating when stimulated at a midpoint in the signalling pathway with PMA/I, we investigated whether VZV interfered with upstream receptor signalling to explain the observed inhibition to target stimulation. Comparing mock, VZV exposed, and VZV infected NK cells either unstimulated, or challenged with K562 cells for 2, 5, 10, or 30 mins, we used intracellular flow cytometry to detect phosphorylated signalling molecules involved in the degranulation pathway. Specifically, we examined phosphorylated expression levels of the Src family tyrosine kinase, Lck; the adaptor proteins, linker for activation of T cells (LAT), and SH2 domain-containing leukocyte protein of 76 kDa (SLP-76); the downstream protein kinases, mitogen-activated or extracellular signal-regulated protein kinase kinase 1 and 2 (MEK1/2), and extracellular signal-regulated kinase 1 and 2 (ERK1/2); and signalling inhibitor, Src homology phosphatase 2 (SHP-2). Following the general temporal signalling order outlined in Fig 8A, we observed similar phosphorylation levels of the earliest markers Lck and LAT between mock, exposed and infected NK cells, at the majority of timepoints (Fig 8B). In contrast, however, both exposed and infected NK cells had notably reduced SLP-76 phosphorylation at rest and when stimulated (Figs 8B and 8C and S6A and S6B). Closer examination revealed that the change in phospho–SLP-76 expression with stimulation was not significantly different between mock, exposed and infected NK cells (S6C Fig). There was, however, a significant reduction in phospho–SLP-76 in both exposed and infected NK cells compared to mock (S6D Fig), indicating that VZV induces a depletion of basal phospho–SLP-76 which does not recover with stimulation.

Downstream we found variable phosphorylation of MEK1/2 but increased basal phosphorylation of ERK1/2 in exposed, and especially infected, NK cells (Fig 8B and 8D). This is consistent with previous reports that the VZV ORF12 protein present in the tegument of VZV virions triggers phosphorylation of ERK1/2 which promotes viral replication and cell survival [30]. We also examined expression of inhibitory SHP-2, but did not observe any distinct differences in levels of phosphorylation between mock, exposed and infected NK cells (Fig 8B). Overall, the data revealed alterations in the degranulation signalling pathway with reduced phospho–SLP-76 in VZV cultured NK cells, as well as a basal upregulation of ERK1/2 phosphorylation.

## Discussion

We have identified a previously unreported strategy employed by VZV to powerfully inhibit NK cell function. Following co-culture with VZV infected epithelial cell inoculum, NK cells were rendered unresponsive to target cell stimulation, exhibiting abrogated cytokine production and degranulation, which correlated with a dramatic reduction in target cell lysis. NK cells were still capable of degranulating when stimulated with PMA/I and maintained relatively stable surface receptor expression, suggesting that VZV paralysed NK cells from responding to target cells. Recently it was observed that patients with varicella displayed significantly lower levels of serum granulysin (a marker of NK cell activity) compared to healthy controls, despite maintained numbers of circulating NK cells [31]. Similarly, a previous study of herpes zoster patients during the acute phase of disease found impaired NK cell activity against K562 targets [32]. These clinical observations lend support to our *in vitro* demonstration of inhibited NK cell function following VZV co-culture, which provides new insights into understanding how VZV interacts with, and evades, the host immune response.

The observation that VZV cultured NK cells degranulated upon PMA/I stimulation indicated that while the NK cells did not respond to target cells, they still retained the capacity and viability to release cytotoxic granules. Cumulatively, PMA/I treatment leads to degranulation through stimulation of protein kinase C-θ (PKC-θ) by PMA, and mobilisation of calcium induced by ionomycin [22]. Unaffected degranulation in VZV cultured NK cells stimulated with PMA/I thus suggested that the point of inhibition was upstream of PMA/I action. In correlation of this finding, examination of key signalling molecules in the NK cell degranulation pathway demonstrated perturbations following VZV co-culture. Notably, in both infected and exposed NK cells we observed a decrease in the level of phosphorylated SLP-76– an adaptor protein central to NK cell activation and degranulation in several pathways of receptor signalling, including through synergistic NKG2D, 2B4 and DNAM-1 stimulation [33]. It was possible that reduced SLP-76 phosphorylation was the result of increased antagonistic phosphatase activity, however when we compared mock and VZV cultured NK cells we did not observe any difference in the level of phospho–SHP-2, which has been previously identified to negatively regulate NK cell cytolytic and cytokine responses [34]. However, it is possible that the recruitment of SHP-2 may be altered with infection, or that VZV induces other unexamined inhibitory phosphatases that have been implicated in suppressing NK cell activity, such as SHP-1 [35]. Given the changes in phosphorylated signalling molecules that we observed, further examination of NK cell signalling following VZV co-culture could shed light on whether modulation of signalling plays a causative role in the inhibition of NK cell function by VZV.

Assays with HSV-1 revealed hampered NK cell responses to target cell stimulation in both HSV-1 infected and exposed NK cells, which was comparable to the phenotype observed with VZV. This finding supports two earlier studies which observed inhibition of NK cell activity following direct contact with HSV infected cells [27, 36]. More recently, HSV has also been characterised to inhibit lymphocyte activity in both cytotoxic T lymphocytes (CTLs) and invariant NKT cells [37, 38]. In these lymphocytes, direct co-culture with HSV led to impaired T cell receptor (TCR) signalling, paralleling the reduced phosphorylation observed in our VZV cultured NK cells when stimulated with K562 cells. Furthermore, it has been found that the U_S_3 protein kinase of HSV-1 partially mediates this inhibition of TCR signalling through interfering with the activation of key adaptor protein LAT [39]. As a closely related alphaherpesvirus, VZV encodes a homologue of U_S_3– ORF66 protein kinase. However, investigation of NK cells cultured with a mutant virus unable to express ORF66 continued to display inhibited degranulation against K562 targets (S7 Fig), indicating that despite similar phenotypes of functional inhibition, VZV appears to elicit its inhibitory effect through an alternative mechanism to that previously identified for HSV-1 inhibition of CTLs. It would be of interest in future to also investigate whether the inhibition of NK cell function by HSV-1 occurs through the viral strategy characterised for CTL inactivation.

NK cells cultured with VZV and HSV-1 were similarly inhibited in their ability to produce cytokines in response to target cell stimulation, implying that they were paralysed from responding. However, there were clear differences between the two viruses when NK cells were stimulated with PMA/I, revealing viral interference with cytokine production independent of the inhibitory block in target cell responsiveness. As demonstrated in mock NK cells, PMA/I triggers a strong induction of IFN-γ and TNF expression, however IFN-γ production was completely abrogated in HSV-1 infected NK cells, while HSV-1 exposed NK cells were unaffected. Conversely, TNF production was not significantly modulated by HSV-1 culture, indicating that HSV-1 infection of NK cells elicits a specific and potent targeting of IFN-γ production. In contrast to HSV-1 infection, VZV infected NK cells displayed reduced expression of both IFN-γ and TNF when stimulated with PMA/I. Additionally, after 2 days of co-culture, VZV exposed NK cells also exhibited suppressed cytokine production, suggesting VZV can affect the efficiency of cytokine expression through several regulatory or inhibitory methods. Recently it was found that NK cells isolated from patients with herpes zoster exhibited significantly lower IFN-γ secretion compared to control subjects, with severity of skin lesion correlated to the degree of IFN-γ suppression [40], corroborating our findings. Reduced IFN-γ and TNF expression upon PMA/I stimulation indicates that VZV and HSV-1 impact points in the cytokine production pathway that are not shared with degranulation, which was unaffected following activation with PMA/I. While degranulation involves the coordinated exocytosis of pre-formed lytic granules, IFN-γ and TNF are not basally present in NK cells and are only expressed following stimulation [6]. Thus, in the process of cytokine synthesis, there are numerous steps–from transcription to protein expression–that could be the target of viral interference as a method of immune evasion. IFN-γ and TNF are crucial in the anti-viral immune response, with both cytokines able to limit viral spread and replication of VZV [41–44] and HSV-1 [45, 46]. The significance of TNF in controlling VZV infection is further exemplified in recent reports of increased reactivation of VZV in patients on anti-TNF regimens [47]. Localised suppression of IFN-γ and/or TNF production by NK cells would conceivably aid both VZV and HSV-1 in establishing infection in the face of a mounting immune response. The cytokine environment also influences NK cell activity, with cytokines driving further cytokine release, proliferation and enhanced cytotoxicity from NK cells. While our study examined the inhibition of cytokine production by NK cells, future investigation could complement these findings by examining additional regulation of cytokine-mediated NK cell responses by alphaherpesvirus infection.

In considering the loss of NK cell responsiveness to target cells following VZV culture, a likely cause would be downregulation of receptor expression; however, profiling of the NK cell surface phenotype suggested that this was not the case. Functional detection of K562 target cells is characterised to be elicited through several receptors, including NKG2D, NKp46, CD160, killer cell immunoglobulin-like receptors (KIRs), NKG2A, and LFA-1 (CD11a) [24, 25, 48–50]. In this study and our previous work [21], we did not find any of these receptors to be consistently downregulated on both exposed and infected NK cells, which would be required to explain the functional inhibition. Furthermore, crosslinking of surface glycoproteins by PHA did not lead to degranulation in VZV cultured NK cells, indicating a global block in responding to broad receptor stimulation. Extensive examination of NK cell receptor expression additionally provided an opportunity to further characterise the influence of VZV on NK cell surface phenotype, which has hitherto not been investigated besides our previous work [21]. Notably, here we found NKp46 expression to be significantly increased on exposed NK cells, especially when concurrently stimulated with IL-2. This upregulation was a trend shared across the other natural cytotoxicity receptors–NKp44 and NKp30– as well as related DNAM-1 and TACTILE, and the direct apoptosis stimulator TRAIL. These changes imply NK cells are dynamically responding to VZV and increasing receptor expression accordingly. It has been found that NK cell detection of HSV-1 infection *in vitro* is predominantly mediated by synergistic activity of the NCRs [51], and thus it was interesting to note upregulation of these receptors on VZV exposed NK cells. It is yet to be characterised for VZV how NK cells detect VZV infection, and it would be of interest to investigate this further. Additionally, examination of the granule content of NK cells following VZV co-culture would reveal whether VZV also targets granzymes and perforin to interfere with NK cell cytolytic function.

It is remarkable that VZV was capable of potently inhibiting the functional responsiveness of not just infected NK cells, but also exposed NK cells that had not undergone productive viral infection. A possible explanation for this was that a secreted factor was responsible, however NK cells were no longer inhibited when separated from VZV inoculum by a semi-permeable membrane or when treated with supernatant from VZV/PBMC co-cultures, thereby precluding the likelihood that inhibition was cytokine-mediated. An alternative explanation is that the VZV inoculum of infected ARPE-19 epithelial cells is presenting an inhibitory ligand that is instigating an inhibited state in the co-cultured NK cells. While this is feasible, it seems unlikely given that NK cells were not inhibited when cultured with inactivated VZV inoculum or cultured with intact VZV-infected ARPE-19s for only 3 hours before stimulation with K562 targets. Potentially, NK cells in co-culture with VZV inoculum may be activated by the infected ARPE-19s, dampening their responsiveness to subsequent K562 stimulation. We have previously shown however that NK cells cultured with VZV infected targets for 5 hours do not display enhanced activation compared to culture with mock targets [17]. Additionally, NK cells were highly responsive (by degranulation and activation) to K562 challenge following co-culture with UV-irradiated or formaldehyde-fixed VZV inoculum, suggesting that engagement with the surface of VZV-infected cells alone does not inhibit NK cells from responding.

Our findings demonstrate that NK cell responses are potently inhibited at 1 and 2 days post co-culture with VZV. Given the prolonged incubation period of VZV during primary infection, studies investigating the extended effects of VZV infection on NK cell function would provide insights into the role of this inhibitory mechanism in viral spread and pathogenesis. The capacity of VZV to functionally inhibit immune cells through direct contact has been previously observed with plasmacytoid dendritic cells, where VZV co-culture blocked stimulated secretion of IFN-α [52]. VZV efficiently infects NK cells through cell-to-cell spread of virus [21], however the exact process of viral spread within lymphocytes has not been fully elucidated. For HSV-1, there is evidence to suggest that infection of T cells occurs through a virological synapse [53]–similar to that which has been characterised for HIV. It is thus possible that in our co-culture system, a similar method of viral spread is occurring with virus entering all NK cells but only continuing to productive infection in a subpopulation of cells. This fits with our observation that the percentage of infected NK cells (as determined by surface VZV gE:gI detection) does not significantly increase from 8–48 hpi, implying there is a predetermined pool of permissive NK cells. We consider this hypothesis to best explain the inhibitory phenotype, which is supported by investigation of HSV-1 inactivation of T cell function, where it was established that inhibition was mediated by virus entry without the need for viral replication, postulating that a virion component was responsible [38]. Our experiments showing that NK cell function is also potently blocked in infected and exposed NK cells co-cultured with cell-free VZV additionally support a model of inhibition mediated directly by the virus.

We have characterised a powerful strategy of immune evasion for VZV, revealing that VZV elicits a profound inhibition of NK cells, paralysing their ability to functionally respond to target cells. We demonstrate a similar inhibitory phenotype elicited by HSV-1, and our findings advance the developing literature uncovering pathogen inhibition of NK cell function. Previous studies have found that the major envelope protein E2 of hepatitis C virus is able to inhibit various NK cell responses through binding of CD81 [54, 55], and similarly the secreted E3/49K protein of adenovirus can suppress functional NK cell signalling through CD45 ligation [56]. Influenza A virus is also capable of reducing NK cell cytotoxicity through direct interactions with haemagglutinin [57], while vaccinia virus mediates inhibition of NK cell cytotoxic function through non-productive infection [58]. These immunosuppression strategies are not a phenomenon unique to viruses, however, with the parasite *Toxoplasma gondii* also downregulating NK cell function following infection [59], as well as several bacterial toxins reported to directly inhibit NK cell activity [60–65]. Cumulatively, these studies, along with our findings presented here, shed light on a common approach–but with distinct mechanisms–utilised by pathogens to restrict effective NK cell function. Elucidating specifically how VZV inhibits NK cells may allow exploitation of this knowledge in therapeutic settings where aberrant NK cell activation can cause immunopathology, such as in certain autoimmune diseases [66], graft-versus-host-disease [67], and transplant rejection [68].

## Materials and methods

### Cells

PBMCs were isolated from healthy human donor buffy coats (provided by the Australian Red Cross Blood Service) by density gradient centrifugation with Ficoll-Paque PLUS (GE Healthcare) and resuspended in complete RPMI medium (RPMI 1640 with L-glutamine [Lonza] supplemented with 10% human serum [Sigma-Aldrich]). CD56^+^ lymphocytes were isolated from PBMCs by MACS positive selection using CD56 MicroBeads, following manufacturer’s protocol (Miltenyi Biotech), and subsequently resuspended in complete RPMI medium. For isolation of NK cells by FACS sorting, CD56^+^-selected lymphocytes were stained with fluorochrome-conjugated antibodies in FACS buffer, and sorted to >95% purity using a FACSAria IIu (BD Biosciences).

The ARPE-19 epithelial cell line, human foreskin fibroblasts (HFFs), and the Vero cell line (all ATCC) were maintained in complete DMEM medium (DMEM with 4.5 g/L glucose and L-glutamine [Lonza] supplemented with 10% foetal calf serum [FCS; Sigma-Aldrich] and penicillin streptomycin [Thermo Fisher Scientific]). K562 cells (ATCC) were maintained in complete RPMI/FCS medium (RPMI 1640 with L-glutamine [Lonza] supplemented with 10% FCS and penicillin streptomycin).

### Viruses

VZV-S, VZV rOka (rOka), and VZV rOka-ORF66S (ORF66S), which is unable to express the ORF66 protein by insertion of a stop codon [69] (all kindly provided by Ann Arvin, Stanford University), were passaged in ARPE-19 cells. Cell-free VZV was generated using HFFs infected with VZV pOka (Ann Arvin, Stanford University) for two days. Mock cell-free was generated in parallel using uninfected HFFs. For cell-free preparations, cells were harvested in cold PBS and then cell pellets were resuspended in PSGC buffer (0.5 g sodium glutamate, 25 g sucrose, 450 ml PBS, autoclaved and then supplemented with 10% FCS). Samples were freeze thawed, sonicated for 3x 30 second intervals with 30 second breaks (Sigma Aldrich Ultrasonic Processor 130W 20 kHz), and centrifuged at 3,000 g for 15 mins. Suspensions were stored at –80°C, and titred on HFFs. HSV-1 pICP47_eGC (HSV-1 GFP-pICP47), HSV-1 pICP6_eGC (HSV-1 GFP-pICP6) and HSV-1 pgB_eGC (HSV-1 GFP-pgB) [28] were grown and titred on Vero cells.

### Co-culture of immune cells with virus

For inoculum input, ARPE-19 cells infected with VZV at a CPE of 2+ to 3+ (approximately 50–75% of cells showing altered morphology) were used. For HSV-1 experiments, ARPE-19 cells were infected with HSV-1 at a multiplicity of infection (MOI) of 0.5 one day prior. Using a cell-associated method of infection, infected or uninfected (mock) inoculum cells were obtained by trypsinisation and combined with PBMCs at a 1:2–1:5 ratio (inoculum to PBMC) in complete RPMI medium in a 12-well plate (or 24-well plate when using CD56^+^-selected lymphocytes). Cell-free VZV infections were performed at an MOI of 0.01–0.1 using 2 x 10^6^ PBMCs in 1 ml of complete RPMI medium in 12-well plates. For all infection methods, plates were subsequently spinoculated at 150 g for 15 mins at room temperature and then cultured at 37°C with 5% CO_2_ for 1 or 2 days, as specified.

For Transwell experiments, inoculum cells were seeded into the bottom chamber with PBMCs above separated by a 0.4 αm pore polycarbonate Transwell membrane (Corning). For experiments with supernatant-treated PBMCs, supernatants were harvested from PBMCs co-cultured with mock or VZV inoculum for 1 day, centrifuged at 460 g to remove cells, and stored at -80°C. Subsequently, supernatants were defrosted and diluted 1:2 with fresh complete RPMI medium before addition to PBMCs for 1 day. For UV-irradiated inoculum experiments, the inoculum cells were seeded into a 12-well plate and irradiated with 1 μJ/cm^2^ (CL-1000 ultraviolet crosslinker) before the addition of PBMCs and spinoculation, as specified above. For fixed inoculum experiments, the inoculum cells were seeded into 12-well plates and allowed to adhere. One day later, monolayers were gently washed with PBS and fixed with 200 μl 1% formaldehyde for 15 mins at room temperature. Monolayers were then washed 3x with PBS before the addition of PBMCs and spinoculation, per the standard protocol outlined.

### Stimulation of NK cells

PBMC co-cultures were stimulated with either K562 target cells at a 1:5 ratio (K562 to PBMC), 100 ng/ml PMA and 1 μg/ml ionomycin (Sigma-Aldrich; kindly provided by Thomas Ashhurst, The University of Sydney), or 10 μg/ml PHA (Sigma-Aldrich). For IL-2 alone and K562 stimulations (unless specified otherwise), PBMCs were first stimulated overnight with 200 U/ml human IL-2 IS (“Improved Sequence”) (Miltenyi Biotec). Stimuli were added direct to PBMC co-cultures in tissue culture plates and incubated at 37°C with 5% CO_2_ for 5 hours, with monensin (BD GolgiStop; BD Biosciences) added for the last 4 hours. For detection of CD107a, a fluorescently-conjugated anti-CD107a antibody (or matched isotype control antibody) was added for the duration of stimulation. All stimulations were performed in technical duplicate. PBMCs were subsequently harvested for flow cytometry analysis.

### Antibodies

For flow cytometry, fluorochrome-conjugated antibodies against the following antigens were used: CD56 (clone NCAM16.2; conjugated to BV605) (B159; APC), CD3 (SK7; BUV395) (HIT3a; PE), CD107a (H4A3; APC), IFN-γ (B27; PerCP/Cy5.5), TNF (MAb11; PE), CD69 (FN50; BV421 and BV711), CD11a (HI111; BV711), NKG2D (149810; PE-CF594), NKp30 (p30-15; BV421), TACTILE (6F9; BV711), TRAIL (RIK-2; BV421), BTLA (J168-540; PE-CF594), Phospho-Lck (4/LCK-Y505; AF647), Phospho-LAT (J96-1238.58.93; PE), Phospho-SLP-76 (J141-668.36.58; PE), Phospho-MEK1/2 (O24-836; PE), Phospho-ERK1/2 (20A; BV421), Phospho-SHP-2 (L99-921; AF647) (all BD Biosciences), CD160 (BY55; PE/Cy7), NKp46 (9E2; PE/Cy7),NKp44 (P44-8; PE), DNAM-1 (11A8; APC), 2B4 (C1.7; PerCP/Cy5.5), NKp80 (5D12; PE) (all BioLegend), NKG2C (134591; APC) (R&D Systems), and VZV gE:gI (SG1-1; conjugated in-house to DyLight 488) (Meridian Life Science). Matched isotype control antibodies were also used where appropriate.

### Flow cytometry

PBMCs were collected and stained with Zombie NIR fixable viability dye (BioLegend), according to manufacturer’s protocol. Cells were then resuspended in FACS buffer (PBS supplemented with 1% FCS and 10 mM EDTA) with antibodies at 4°C for at least 30 mins. Cells were next fixed in 1% formaldehyde (Cytofix; BD Biosciences) at 4°C for at least 15 mins. For intracellular staining, cells were then permeabilised by washing twice in BD Perm/Wash Buffer (BD Biosciences), staining for intracellular markers in Perm/Wash at 4°C for at least 30 mins, and concluding with 3 washes in Perm/Wash. Stained cells were resuspended in FACS buffer and acquired on an LSR-II cytometer (BD Biosciences).

### Phosflow flow cytometry

For examining phosphorylation of signalling markers with K562 stimulation, PBMC co-cultures plus IL-2 were established as described above. One day pi PBMCs were collected and either left unstimulated, or combined with K562 target cells at a 1:2 ratio (K562 to PBMC) for 2, 5, 10 or 30 mins on a rocking platform at 37°C. Following the BD Biosciences Phosflow protocol, activation was terminated by immediately fixing with 1 ml warmed 4% formaldehyde (Cytofix; BD Biosciences) for 10 mins at 37°C. Cells were then washed with FACS buffer and permeabilised by slowly adding 1 ml Perm Buffer III (pre-cooled to -20°C) (BD Biosciences) while vortexing, then incubated on ice for 30 mins. Cells were subsequently washed before resuspension in FACS buffer with antibodies at room temperature for 60 mins. Finally, cells were washed again and suspended in FACS buffer for acquisition on an LSR-II cytometer (BD Biosciences).

### Flow cytometry data analysis

Flow cytometry data were analysed with FlowJo software (versions 10.0.7 and 10.2; Tree Star). All data depicted are gated on live cells, as determined by Zombie NIR fixable viability dye staining. Where functional activity was measured (CD107a, IFN-γ and TNF expression) values were determined as the mean of two technical duplicates. Where specified, specific degranulation was calculated as degranulation (CD107a^+^) against K562 targets minus background degranulation (CD107a^+^) when unstimulated. For presentation of polyfunctional responses, flow cytometry data was visualised using SPICE software (Version 6.0) [70]. Heatmaps and dendrograms were generated using the matrix visualisation and analysis software Morpheus (https://software.broadinstitute.org/morpheus).

### Calcein-AM release assay

NK cell cytolytic function was assessed using the calcein-AM release microassay as previously described [71]. Briefly, PBMC co-cultures plus IL-2 were established as described above. One day pi PBMCs were collected and NK cells (CD3^–^CD56^+^) isolated by FACS sorting, and rested overnight in complete RPMI medium plus IL-2. Using a 96-well V-bottom plate, varying numbers of NK cells were combined with 5 x 10^2^ K562 target cells labelled prior with 15 μM calcein-AM (Sigma-Aldrich), to achieve NK cell effector to target cell ratios of 20:1, 10:1, 5:1 and 2.5:1, and performed in triplicate. To control for spontaneous release of dye, labelled K562s were cultured in parallel without NK cells. In order to calculate percentage of target cell lysis, maximum release was determined by parallel culture of labelled K562s lysed with 2% Triton X-100 (Sigma-Aldrich). Controls were performed in 6 technical replicates. The plate was briefly centrifuged and then incubated at 37°C with 5% CO_2_ for 4 hours. Subsequently cells were pelleted by centrifugation and supernatants harvested and transferred to a 96-well flat-bottom plate. Fluorescence was measured using a TECAN Infinite M1000 Pro. Percentage of target cell lysis (% specific release) was calculated as: ([experimental release–spontaneous release] / [maximum release–spontaneous release]) x 100.

### Statistics

Statistical analysis was performed with GraphPad Prism (version 7; GraphPad Software).

### Ethics statement

All human blood work was performed in accordance with The University of Sydney ethics approval and all donors provided written informed consent.

## Supporting information

S1 Fig NKG2D and NKp46 cell surface expression following VZV culture. PBMCs were mock cultured, exposed to VZV, or VZV infected untreated or with 200 U/ml IL-2 for 1 day. NK cells (viable CD3^–^CD56^+^ cells) were assessed by flow cytometry for cell surface expression of NKG2D (A) and NKp46 (B). Graphs show fold change over mock in median fluorescence intensity (MFI) (n = 9). Symbols represent individual donors. Dotted line at y = 1 indicates point of variance from mock. Statistical analysis performed compared to mock. *P < 0.05, **P < 0.01 (repeated measures one-way ANOVA with Dunnett’s correction).
(TIF)

S2 Fig Profiling of cell surface receptor phenotype of VZV cultured CD56^+^-selected NK cells. CD56^+^-selected lymphocytes were mock cultured, exposed to VZV, or VZV infected untreated or with 200 U/ml IL-2 for 1 or 2 days. NK cells (viable CD3^–^CD56^+^ cells) were assessed by flow cytometry for cell surface receptor expression. (A) Heatmaps show receptor expression as measured by percentage positive with hierarchical clustering for 2 donors (denoted 1 and 2) (B). (B) Graphs show fold change over mock in median fluorescence intensity (MFI) for ubiquitously expressed receptors (n = 2). Symbols represent individual donors. Dotted line at y = 1 indicates point of variance from mock. Statistical analysis performed compared to mock. *P < 0.05, ns = not significant (repeated measures two-way ANOVA with Dunnett’s correction).
(TIF)

S3 Fig VZV culture inhibits NK cell degranulation with PHA stimulation. (A) PBMCs were mock cultured, exposed to VZV, or VZV infected for 2 days and stimulated with PHA or left unstimulated. Flow cytometry plots NK cell (viable CD3^–^CD56^+^ cells) degranulation (CD107a^+^), representative of two donors.
(TIF)

S4 Fig Cell-free VZV impairs NK cell function towards K562 cells. PBMCs were cultured with mock or VZV cell-free preparations (MOI 0.01–0.1), or cultured with cell-associated VZV inoculum, for 1 day. (A) Flow cytometry detection of VZV infection (gE:gI^+^) of NK cells. (B & C) Flow cytometry of degranulation (CD107a^+^) of NK cells (viable CD3^–^CD56^+^ cells) cultured with mock or VZV cell-free preparations, and stimulated with K562 cells with IL-2 or left unstimulated. VZV exposed or infected was determined by surface staining for VZV gE:gI. Graph shows frequency of specific degranulation against K562 cells for two donors. Symbols represent individual donors, and grey columns indicate mean.
(TIF)

S5 Fig Inactivation of VZV inoculum eliminates the inhibition of NK cell cytolytic function by VZV. (A & B) PBMCs were cultured with intact mock or VZV inoculum (A) or inoculum monolayers inactivated prior with UV-irradiation (B). After 1 day, PBMCs were challenged with K562 cells with IL-2 or left unstimulated, and analysed by flow cytometry. NK cells (viable CD3^–^CD56^+^ cells) were examined for degranulation (CD107a^+^) (dot plots) and activation (CD69^+^) (histograms). (C) PBMCs were cultured with mock or VZV inoculum monolayers fixed prior with 1% formaldehyde. After 1 day, PBMCs were challenged with K562 cells with IL-2 or left unstimulated, and NK cells (viable CD3^–^CD56^+^ cells) assessed by flow cytometry for degranulation (CD107a^+^) (dot plots) and activation (CD69^+^) (histograms).
(TIF)

S6 Fig VZV culture reduces basal expression of phospho–SLP-76. (A–D) PBMCs were mock cultured, exposed to VZV, or VZV infected in the presence of 200 U/ml IL-2 for 1 day and either left unstimulated or stimulated with K562 cells for 2, 5, 10 or 30 min as specified. Phosphorylation of SLP-76 in NK cells (CD3–CD56+cells) was detected by flow cytometry. (A) Histograms show phospho–SLP-76 expression for NK cells unstimulated and after 10 min stimulation with K562 cells, for two donors. Median fluorescence intensity (MFI) values are indicated on the top left of the histogram. (B) Heatmap of phospho–SLP-76 expression MFI fold increase. (C & D) MFI was analysed as fold change over respective unstimulated values for mock, exposed and infected NK cells (C) or as fold change over mock (D) (n = 3). Symbols represent individual donors, and filled columns indicate mean. Statistical analysis performed comparing differences between conditions (mock, exposed, infected) and between timepoints. ****P < 0.0001, ns = not significant (Repeated measures two-way ANOVA with Geisser-Greenhouse correction, and Dunnett’s multiple comparisons test). E, exposed; I, infected.
(TIF)

S7 Fig VZV ORF66 does not mediate VZV inhibition of NK cell cytolytic function. PBMCs were cultured with mock inoculum or inoculum infected with parental rOka VZV or ORF66S-rOka VZV (ORF66S) for 1 day. PBMCs were stimulated with K562 target cells with IL-2 (A) or PMA/I (B), and NK cells (viable CD3^–^CD56^+^ cells) assessed by flow cytometry for specific degranulation (CD107a^+^). Symbols represent individual donors, and grey columns indicate mean. Data are from two donors (A & B).
(TIF)
