# Supplementary figures and images for: Functional paralysis of human natural killer cells by alphaherpesviruses
Source: PLoS Pathog. 2019 Jun 13;15(6):e1007784. doi: 10.1371/journal.ppat.1007784 (PMC6564036; doi:10.1371/journal.ppat.1007784)

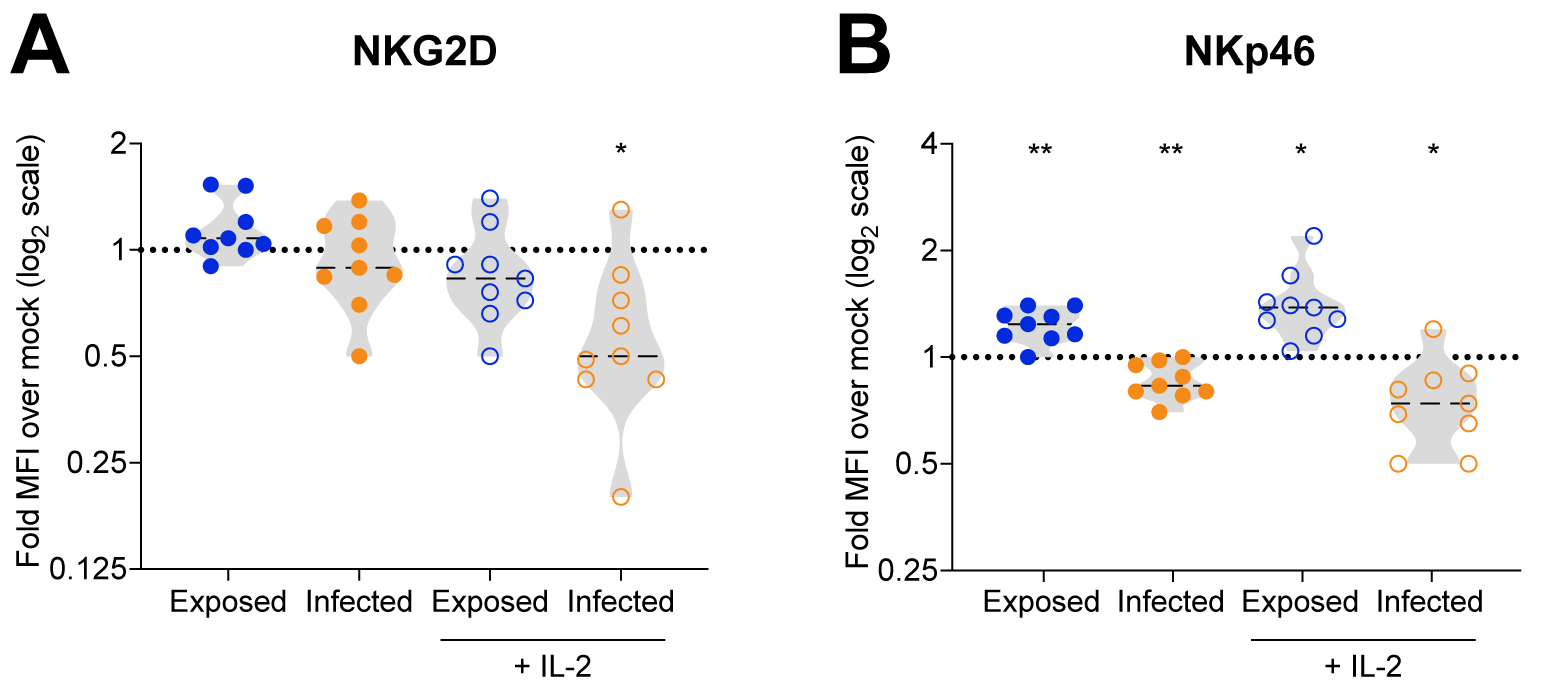

Supplement: S1 Fig — PBMCs were mock cultured, exposed to VZV, or VZV infected untreated or with 200 U/ml IL-2 for 1 day. NK cells (viable CD3–CD56+ cells) were assessed by flow cytometry for cell surface expression of NKG2D (A) and NKp46 (B). Graphs show fold change over mock in median fluorescence intensity (MFI) (n = 9). Symbols represent individual donors. Dotted line at y = 1 indicates point of variance from mock. Statistical analysis performed compared to mock. *P < 0.05, **P < 0.01 (repeated measures one-way ANOVA with Dunnett’s correction). (TIF) [file ppat.1007784.s001.tif]

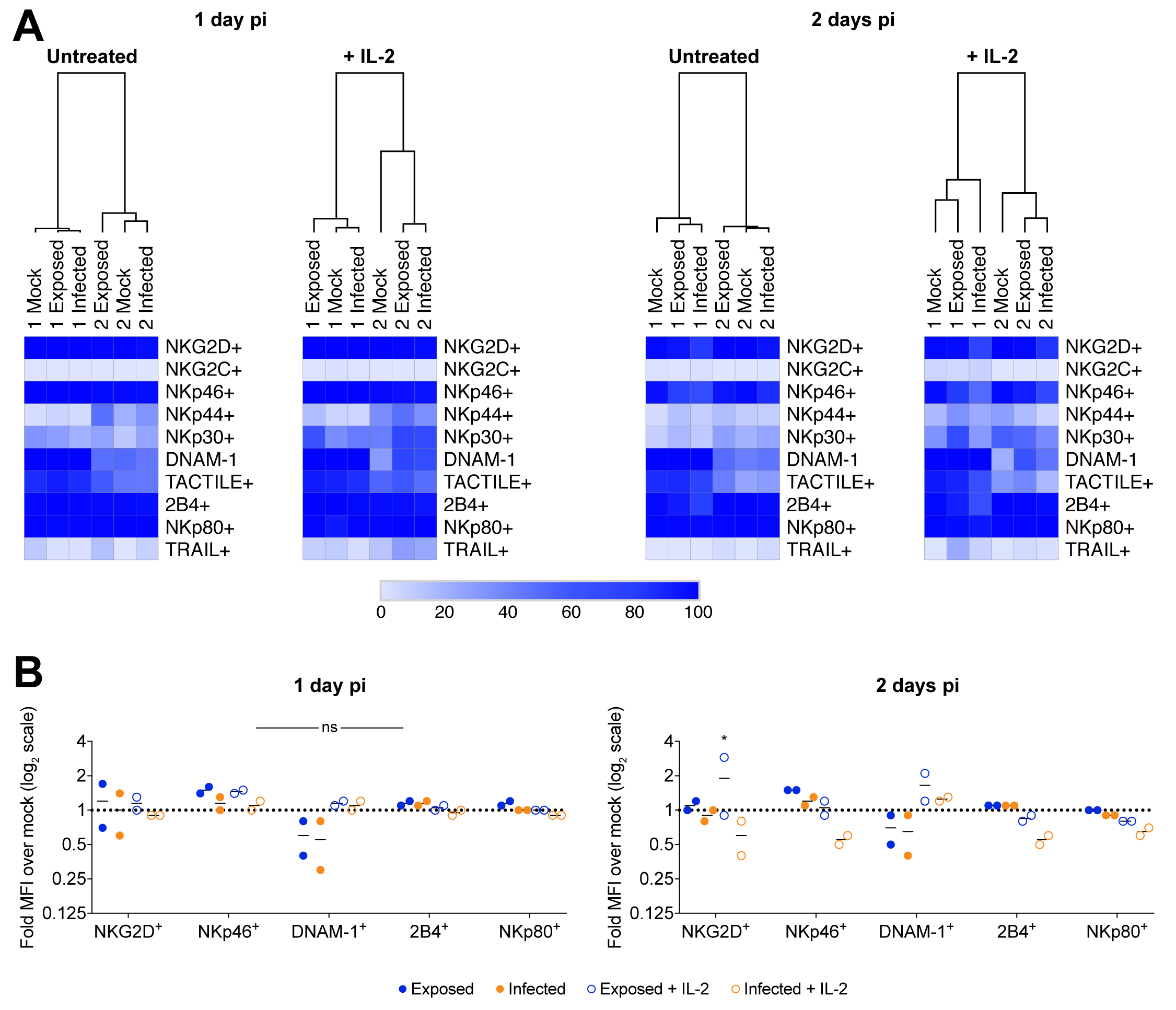

Supplement: S2 Fig — CD56+-selected lymphocytes were mock cultured, exposed to VZV, or VZV infected untreated or with 200 U/ml IL-2 for 1 or 2 days. NK cells (viable CD3–CD56+ cells) were assessed by flow cytometry for cell surface receptor expression. (A) Heatmaps show receptor expression as measured by percentage positive with hierarchical clustering for 2 donors (denoted 1 and 2) (B). (B) Graphs show fold change over mock in median fluorescence intensity (MFI) for ubiquitously expressed receptors (n = 2). Symbols represent individual donors. Dotted line at y = 1 indicates point of variance from mock. Statistical analysis performed compared to mock. *P < 0.05, ns = not significant (repeated measures two-way ANOVA with Dunnett’s correction). (TIF) [file ppat.1007784.s002.tif]

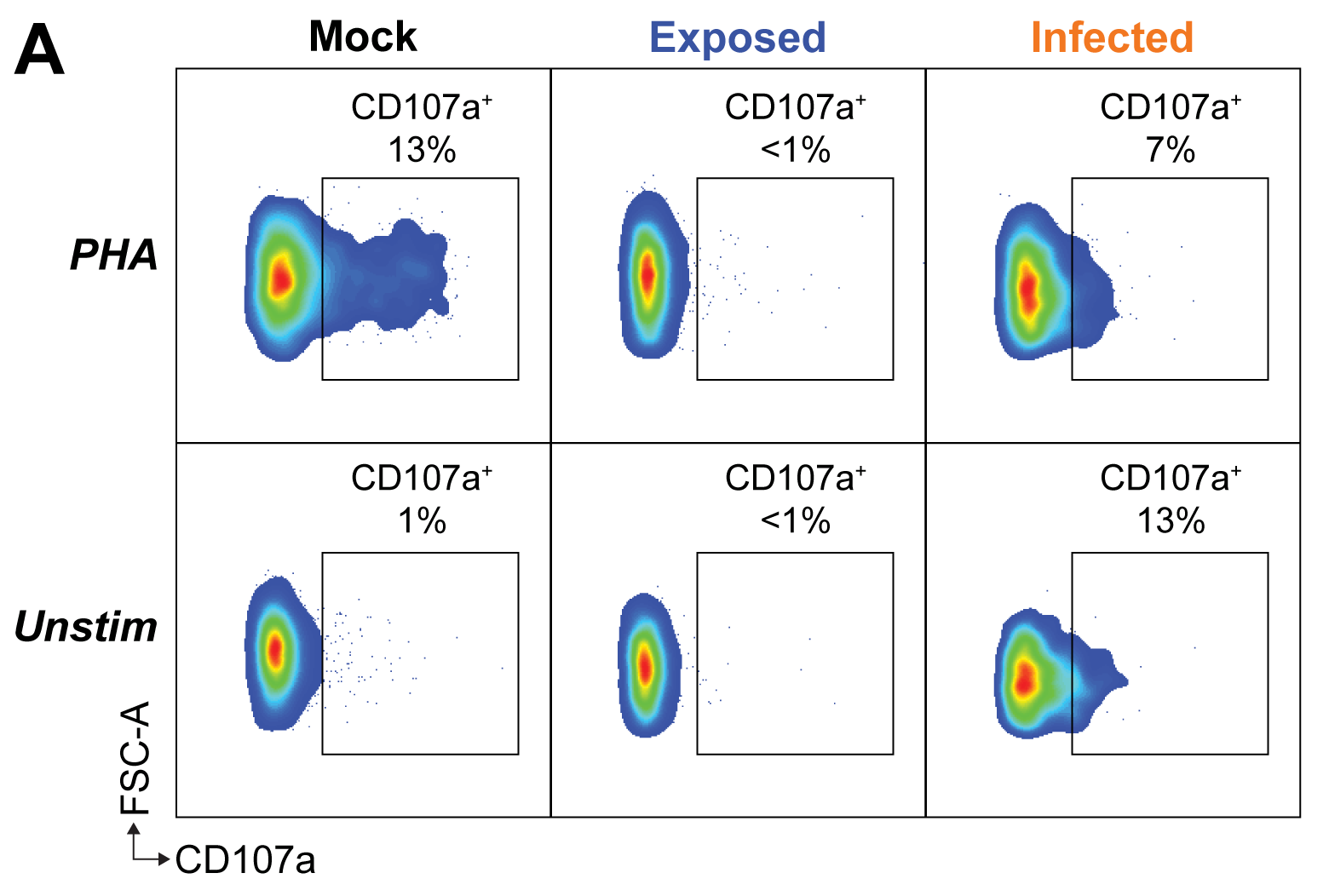

Supplement: S3 Fig — (A) PBMCs were mock cultured, exposed to VZV, or VZV infected for 2 days and stimulated with PHA or left unstimulated. Flow cytometry plots NK cell (viable CD3–CD56+ cells) degranulation (CD107a+), representative of two donors. (TIF) [file ppat.1007784.s003.tif]

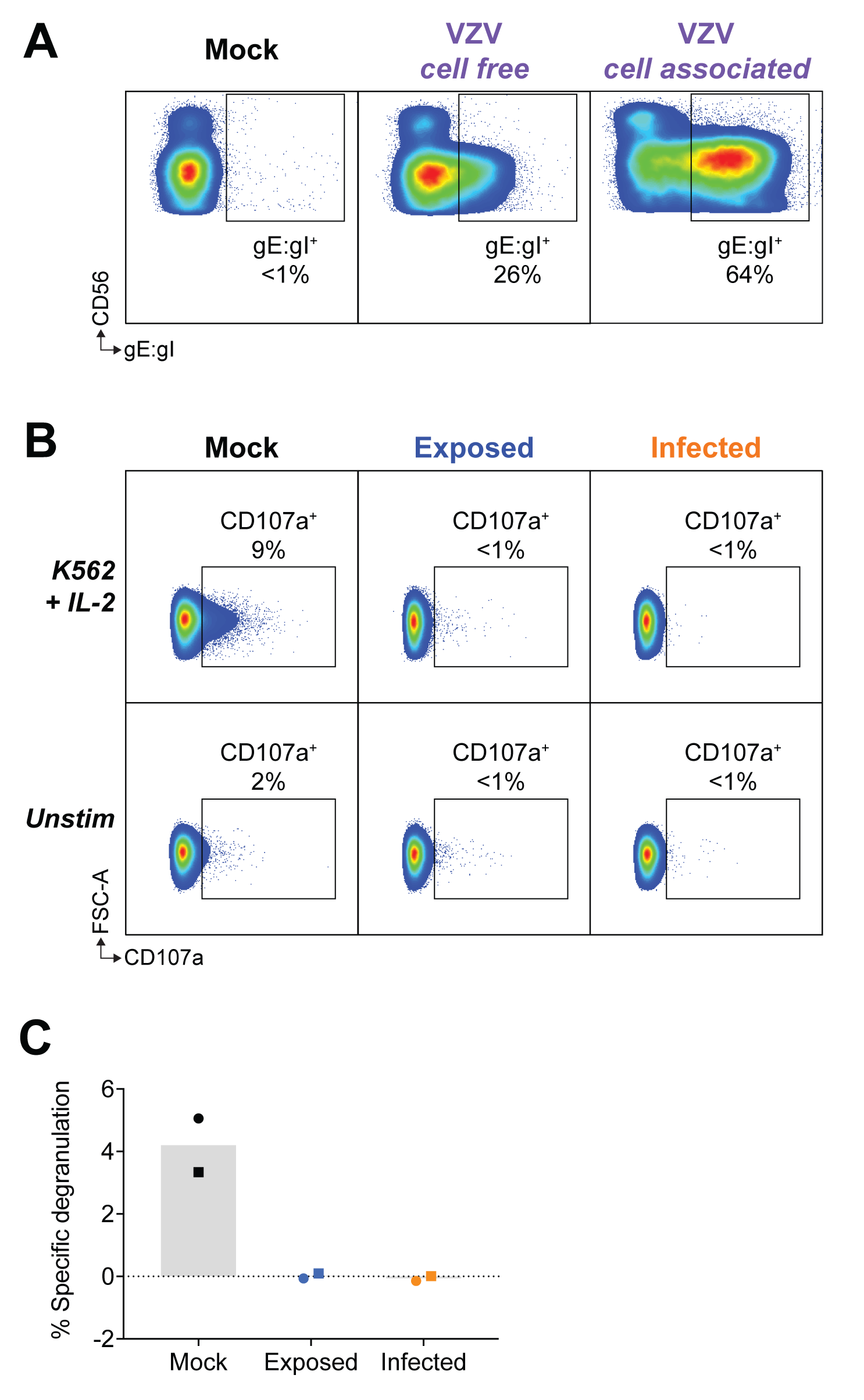

Supplement: S4 Fig — PBMCs were cultured with mock or VZV cell-free preparations (MOI 0.01–0.1), or cultured with cell-associated VZV inoculum, for 1 day. (A) Flow cytometry detection of VZV infection (gE:gI+) of NK cells. (B & C) Flow cytometry of degranulation (CD107a+) of NK cells (viable CD3–CD56+ cells) cultured with mock or VZV cell-free preparations, and stimulated with K562 cells with IL-2 or left unstimulated. VZV exposed or infected was determined by surface staining for VZV gE:gI. Graph shows frequency of specific degranulation against K562 cells for two donors. Symbols represent individual donors, and grey columns indicate mean. (TIF) [file ppat.1007784.s004.tif]

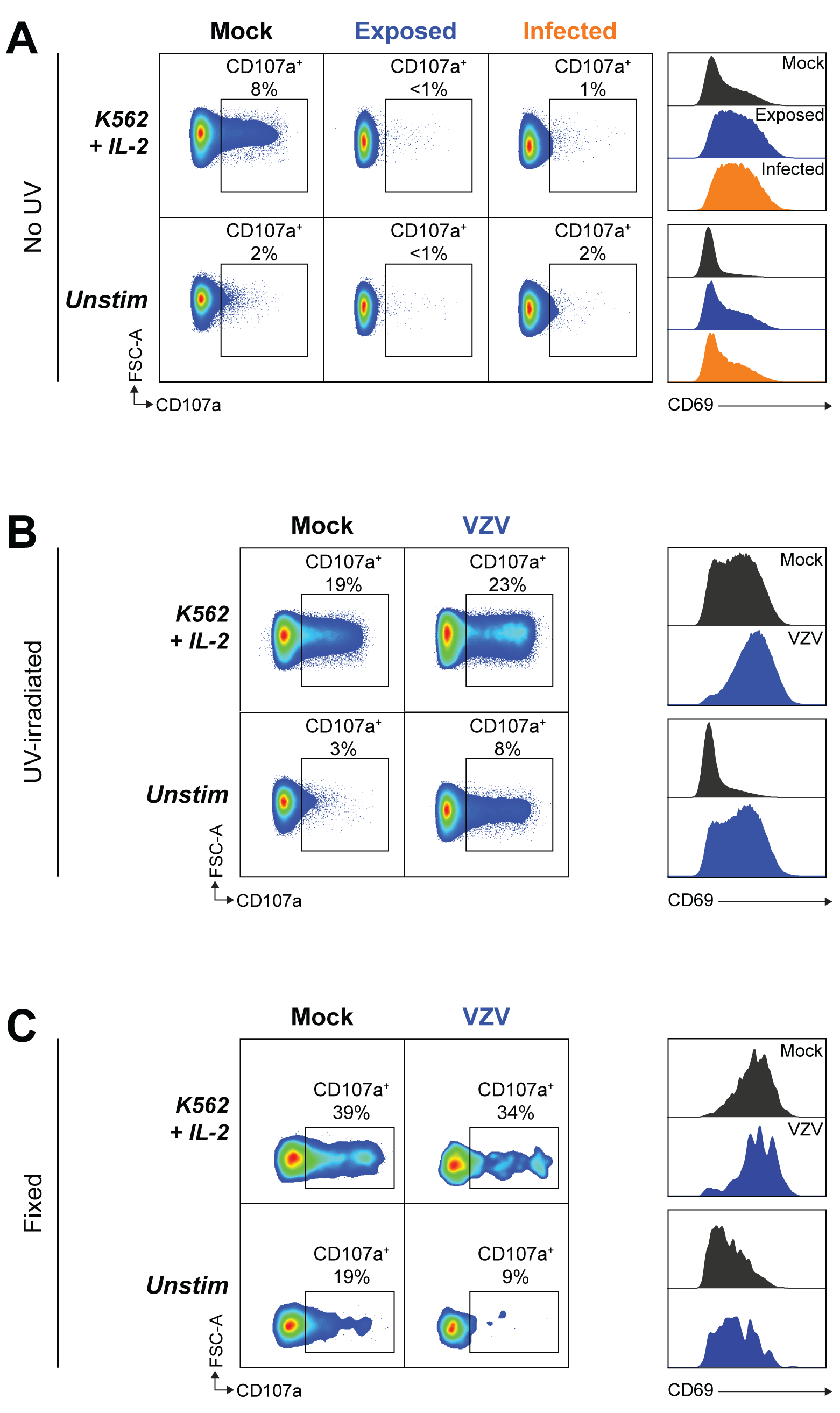

Supplement: S5 Fig — (A & B) PBMCs were cultured with intact mock or VZV inoculum (A) or inoculum monolayers inactivated prior with UV-irradiation (B). After 1 day, PBMCs were challenged with K562 cells with IL-2 or left unstimulated, and analysed by flow cytometry. NK cells (viable CD3–CD56+ cells) were examined for degranulation (CD107a+) (dot plots) and activation (CD69+) (histograms). (C) PBMCs were cultured with mock or VZV inoculum monolayers fixed prior with 1% formaldehyde. After 1 day, PBMCs were challenged with K562 cells with IL-2 or left unstimulated, and NK cells (viable CD3–CD56+ cells) assessed by flow cytometry for degranulation (CD107a+) (dot plots) and activation (CD69+) (histograms). (TIF) [file ppat.1007784.s005.tif]

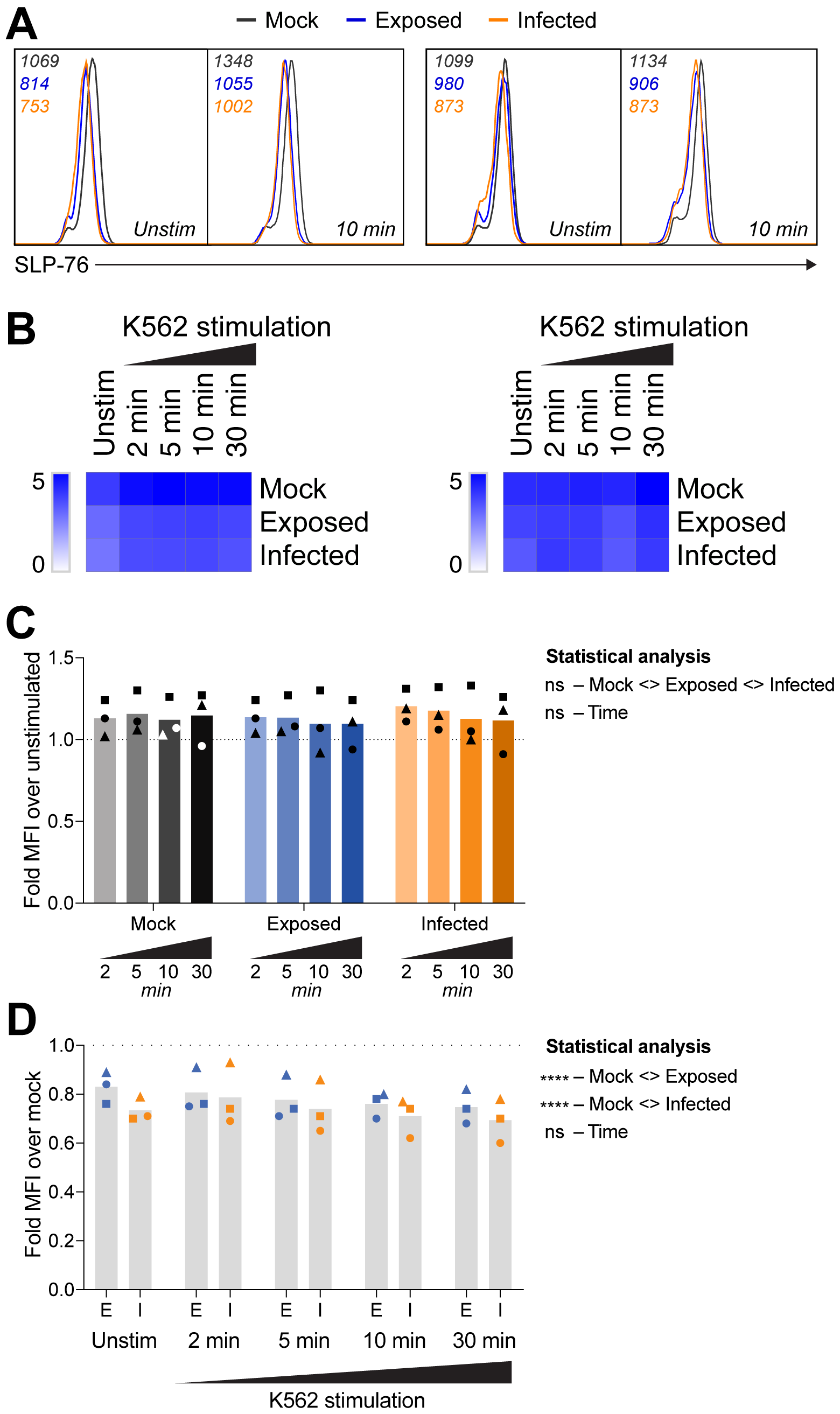

Supplement: S6 Fig — (A–D) PBMCs were mock cultured, exposed to VZV, or VZV infected in the presence of 200 U/ml IL-2 for 1 day and either left unstimulated or stimulated with K562 cells for 2, 5, 10 or 30 min as specified. Phosphorylation of SLP-76 in NK cells (CD3–CD56+cells) was detected by flow cytometry. (A) Histograms show phospho–SLP-76 expression for NK cells unstimulated and after 10 min stimulation with K562 cells, for two donors. Median fluorescence intensity (MFI) values are indicated on the top left of the histogram. (B) Heatmap of phospho–SLP-76 expression MFI fold increase. (C & D) MFI was analysed as fold change over respective unstimulated values for mock, exposed and infected NK cells (C) or as fold change over mock (D) (n = 3). Symbols represent individual donors, and filled columns indicate mean. Statistical analysis performed comparing differences between conditions (mock, exposed, infected) and between timepoints. ****P < 0.0001, ns = not significant (Repeated measures two-way ANOVA with Geisser-Greenhouse correction, and Dunnett’s multiple comparisons test). E, exposed; I, infected. (TIF) [file ppat.1007784.s006.tif]

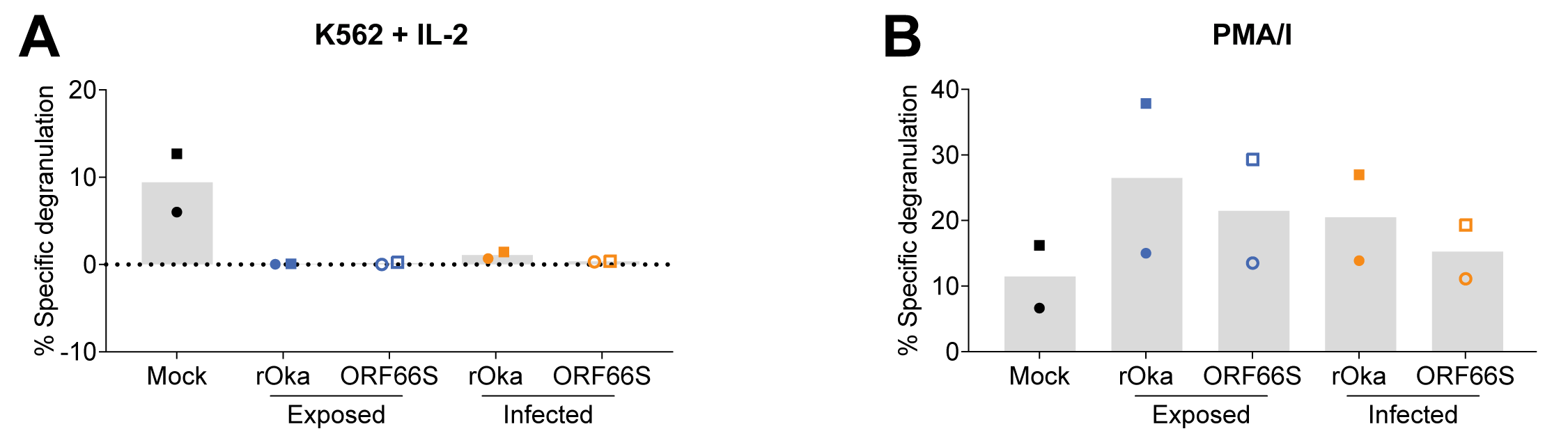

Supplement: S7 Fig — PBMCs were cultured with mock inoculum or inoculum infected with parental rOka VZV or ORF66S-rOka VZV (ORF66S) for 1 day. PBMCs were stimulated with K562 target cells with IL-2 (A) or PMA/I (B), and NK cells (viable CD3–CD56+ cells) assessed by flow cytometry for specific degranulation (CD107a+). Symbols represent individual donors, and grey columns indicate mean. Data are from two donors (A & B). (TIF) [file ppat.1007784.s007.tif]
